# Supplementary material for: Synthetic Lethality between DNA Polymerase Epsilon and RTEL1 in Metazoan DNA Replication
Source: Cell Rep. 2020 May 26;31(8):107675. doi: 10.1016/j.celrep.2020.107675 (PMC7262601; doi:10.1016/j.celrep.2020.107675)
Supplement: Document S1. Figures S1–S5 and Tables S1 and S2 [file mmc1.pdf]

Cell Reports, Volume *31*

Supplemental Information

Synthetic Lethality between DNA Polymerase Epsilon and RTEL1 in Metazoan DNA Replication

Roberto Bellelli, Jillian Youds, Valerie Borel, Jennifer Svendsen, Visnja Pavicic-Kaltenbrunner, and Simon J. Boulton

Supplemental Figure S1

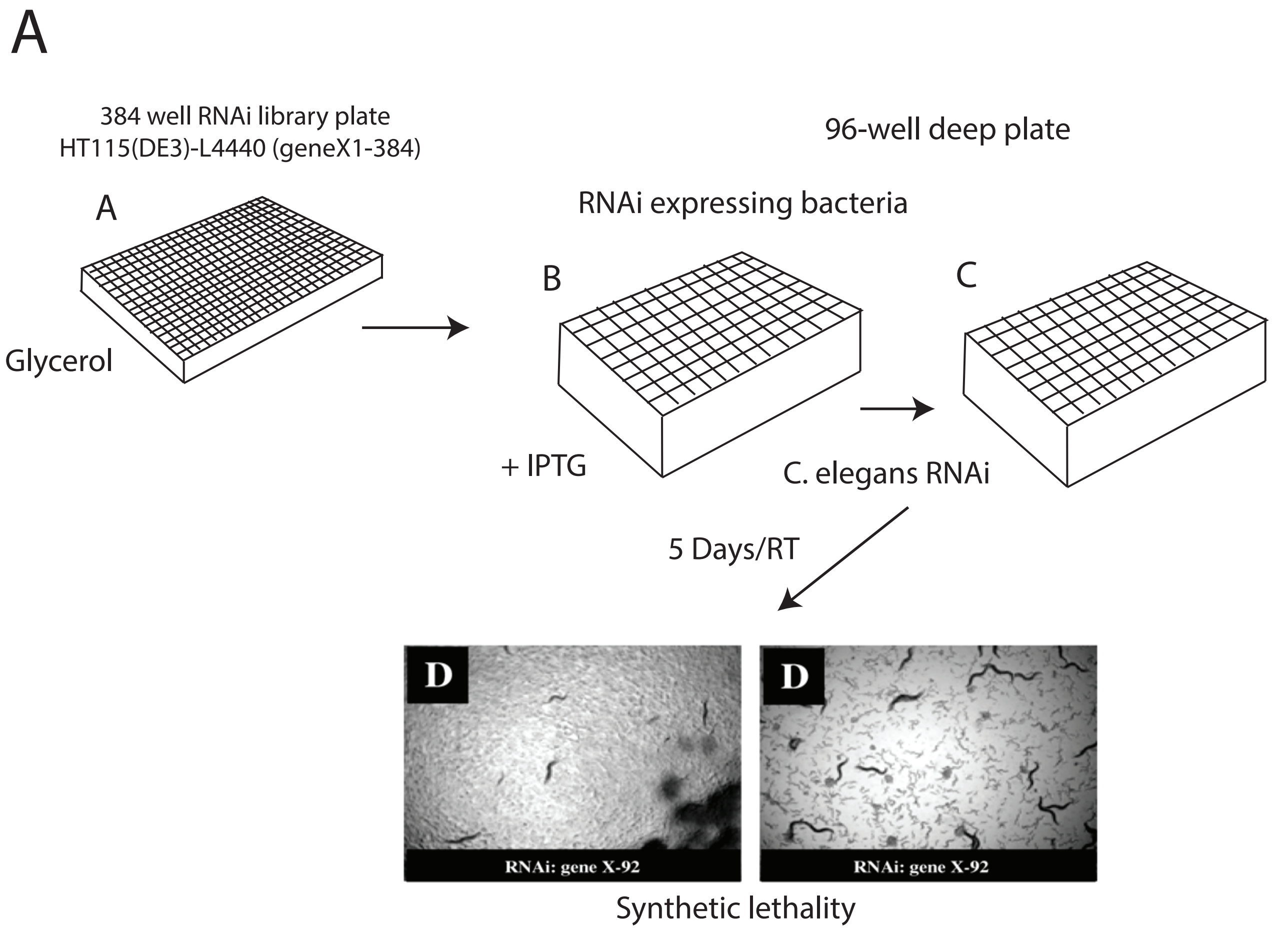

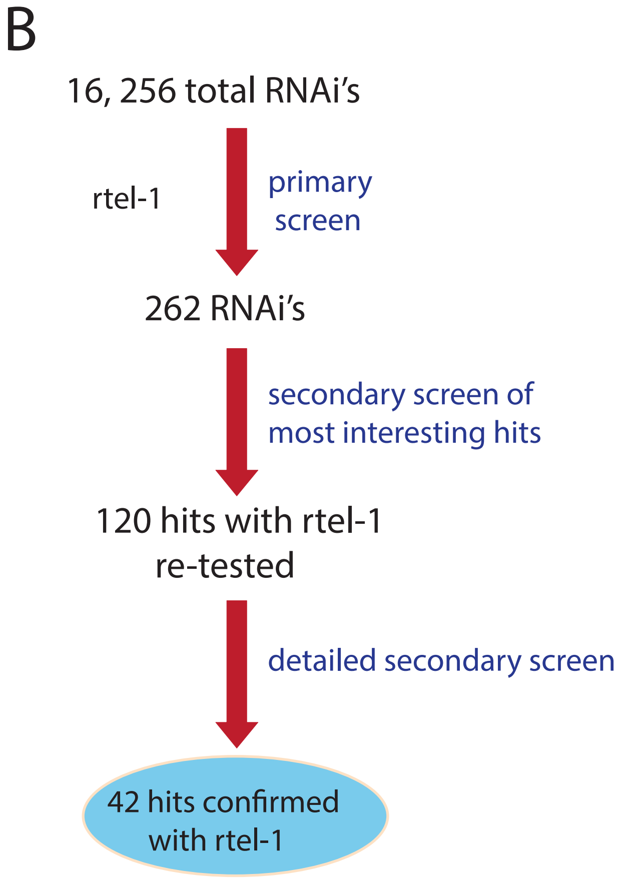

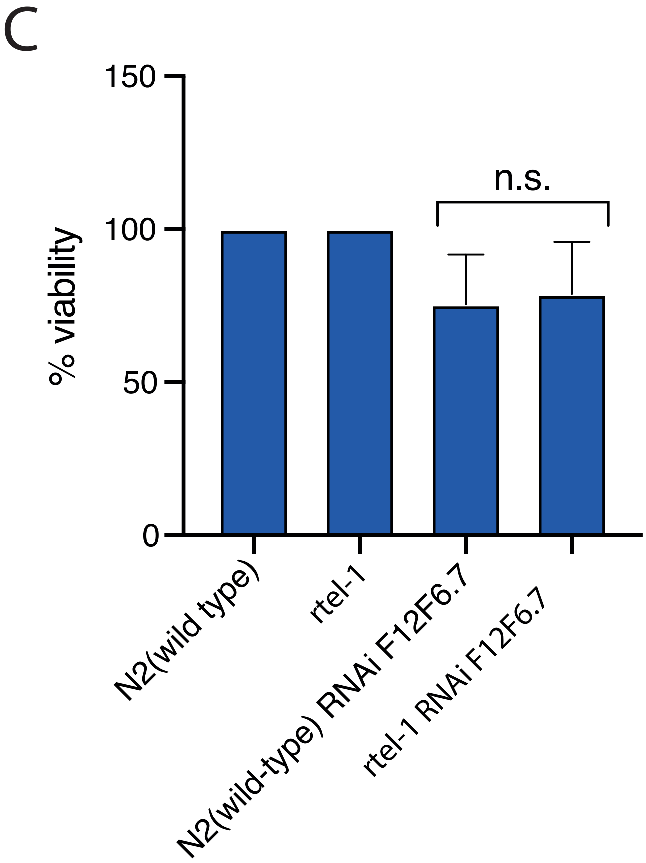

Figure S1 (related to Figure 1). A synthetic viability screening in *rtel-1* mutant C. elegans animals. (A) Representative scheme of the RNAi screening performed in *rtel-1* wt and mutant worms. (B) Diagram showing the sequential primary and secondary RNAi screenings performed in *rtel-1* wt and mutant strains and the confirmed hits (C). Percent viability after feeding either no RNAi or RNAi for *pold-2* (F12F6.7) in wild type or *rtel-1* mutant worms. percent viability is normalized based on untreated wild type or *rtel-1* control animals (n.s. not significant).

Supplemental Figure S2

A

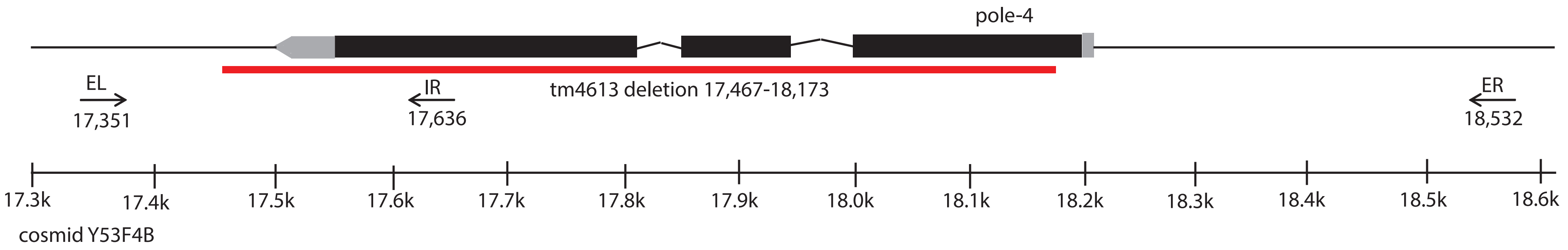

B

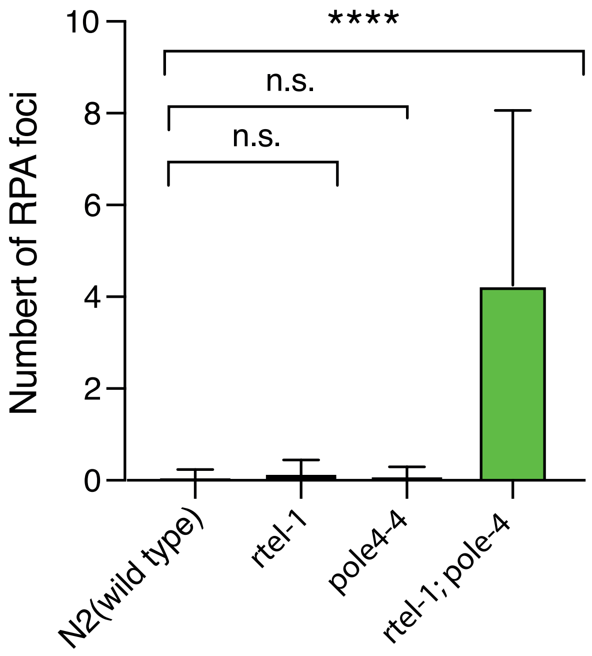

Figure S2 (related to Figure 2). (A) Schematic diagram of C. elegans *Y53F4B.3* (*pole-4*) gene. The genetic deletion identified in the *tm4613* strain is highlighted with a red bar. (B). Bar-graphs showing the average number of RPA foci +/- SD in the indicated genotypes (**** p <0.0001, n.s. not significant).

Supplemental Figure S3

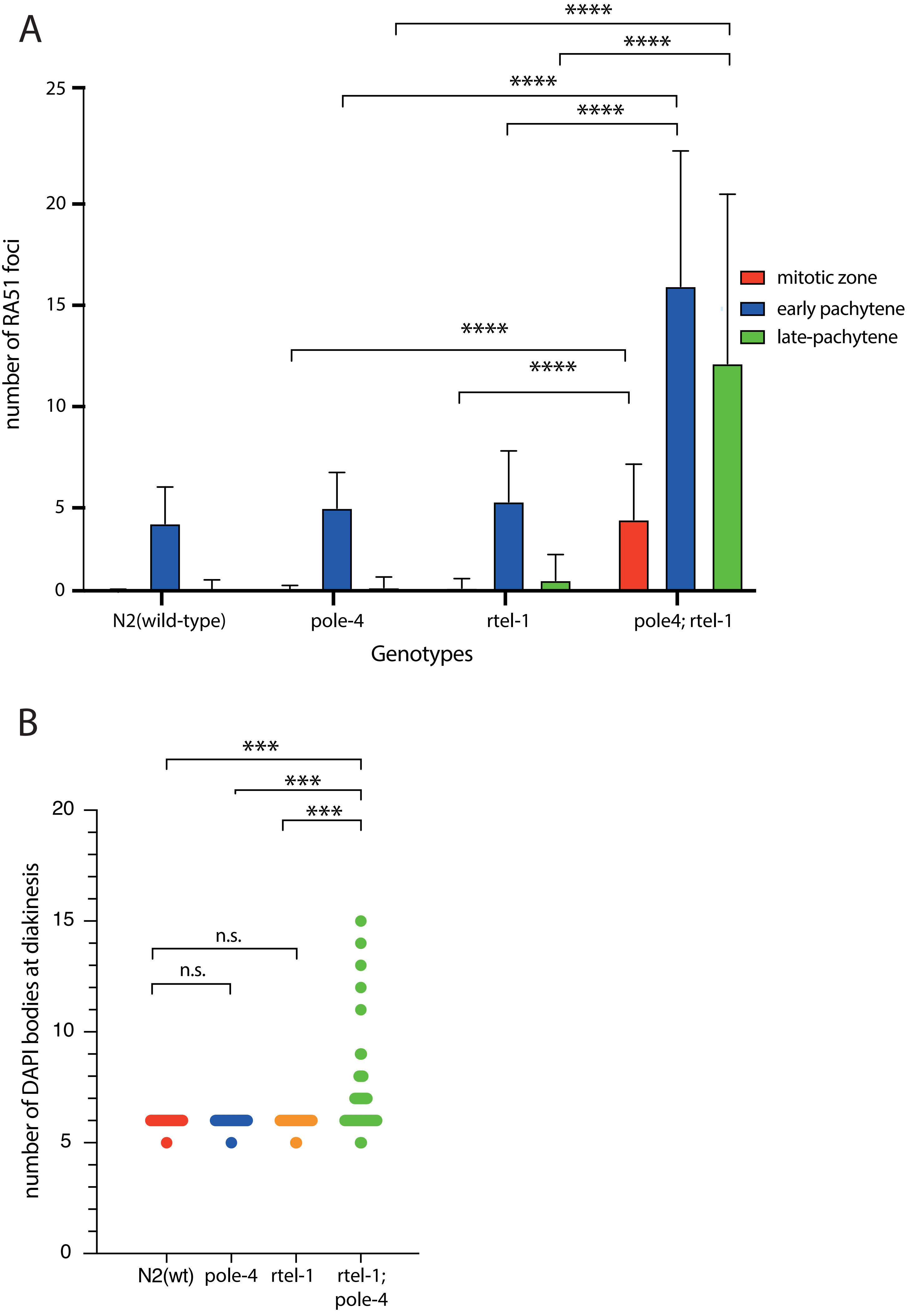

Figure S3 (related to Figure 3). (A) Bar-graphs showing the number of RAD51 foci in the defined zones of the germline in the indicated genotypes (**** $p< 0.0001$). (B). Bar-graphs showing the number of DAPI bodies at diakinesis in the indicated genotypes (*** $p< 0.001$, n.s. not significant).

Supplemental Table S1

Genotype	Brood Size	Percent Survival	Percent Males (of viable)
**wild type**	299 ±	99.6 ± 0.1	0.1 ± 0.1
pole-4 (tm4613)	237 ±	99.8 ± 0.1	0.0 ± 0.0
dog-1 (gk10)	300 ± 28	99.7 ± 0.2	0.1 ± 0.1
rtel-1 (tm1866)	61 ± 16	92.6 ± 3.6	0.0 ± 0.0
mus-81 (tm1937)	114 ± 19	90.1 ± 3.7	0.1 ± 0.1
him-6 (ok412)	221 ± 13	48.3 ± 3.2	11.7 ± 0.8
rcq-5(tm424)	292 ± 32	99.4 ± 1.0	0.1 ± 0.1
rtel-1; pole-4	29 ±	0	N/A
dog-1; pole-4	198 ± 15	91.6 ± 5.1	0.3 ± 0.1
mus-81; pole-4	107 ± 9	96.7 ± 1.0	0.6 ± 0.3
pole-4; him-6	123 ± 15	48.2 ± 3.3	11.7 ± 1.0
pole-4; rcq-5	227 ± 22	99.4 ± 0.2	0.0 ± 0.0
rtel-1 mus-81; pole-4	0	0	N/A
rtel-1; pole-4; rfs-1	20 ± 7	0	N/A

Supplemental Table S1 (related to Figure 4). Genetic interactions of *pole-4*. Brood size indicates the average total number of eggs laid by individual animals. Percent survival indicates the percent of the total brood that survived to adulthood. For each genotype, the progeny of at least 10 parent animals were scored.

Supplemental Table S2

Genotype	Animals Tested	Animals with Deletions	Percent with Deletions
**wild type**	120	0	0
dog-1(gk10)	192	15	7.8
pole-2(RNAi)	120	0	0
dog-1; pole-2(RNAi)	228	18	7.9
rtel-1(tm1866)	92	0	0
pole-4 (tm4613)	96	0	0
rtel-1; pole-4	96	0	0

Supplemental Table S2 (related to Figure 4). Poly-G-tracts are stable in polymerase epsilon mutants and in *rtel-1; pole-4* animals. The presence of G-tract deletions in the genotypes indicated was measured by nested PCR at the G-tract within the *vab-1* gene.

Supplemental Figure S4

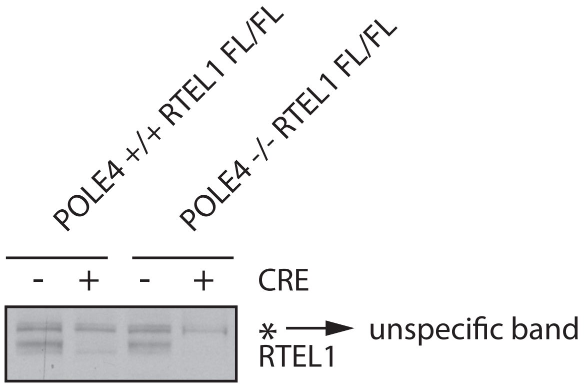

Figure S4 (related to Figure 5). Western blot analysis of RTEL1 expression levels in RTEL1$^{F/F}$ *POLE4*$^{+/+}$ and RTEL1$^{F/F}$ *POLE4*$^{-/-}$ MEFs infected with GFP-CRE or empty GFP. An unspecific band (*) served as loading control.

Supplemental Figure S5

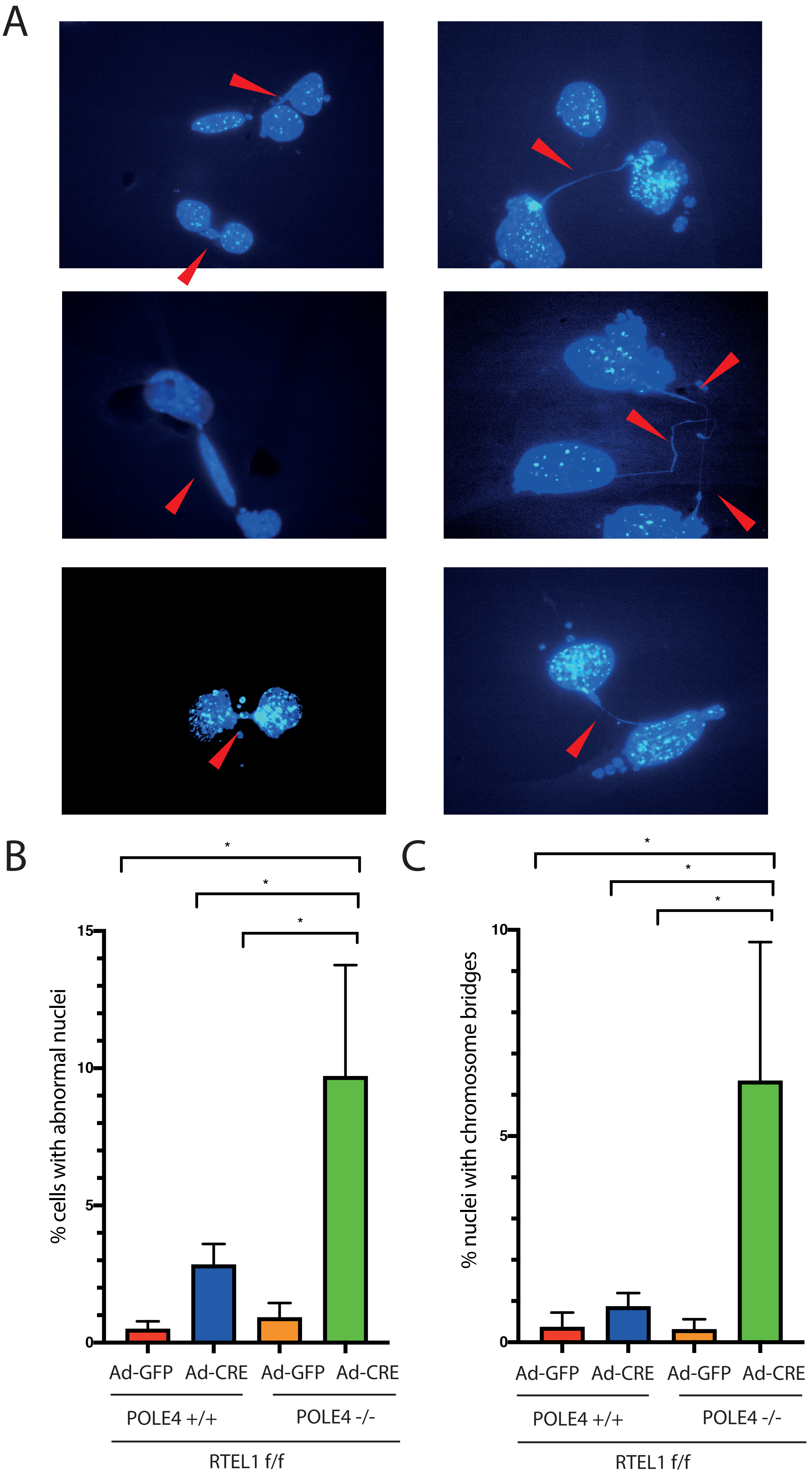

Figure S5 (related to Figure 6). (A) Representative images of nuclear aberrations observed in RTEL1$^{F/F}$ *POLE*$^{-/-}$ MEFs infected with GFP-CRE. Red arrows indicate mitotic bridges. (B) Bar-graphs showing percentage of cells with abnormal nuclear structures from RTEL1$^{F/F}$ *POLE4*$^{+/+}$ and RTEL1$^{F/F}$ *POLE4*$^{-/-}$ infected or not with CRE (* $p< 0.05$). (C) Bar-graphs showing percentage of nuclei with chromosome bridges from RTEL1$^{F/F}$ *POLE4*$^{+/+}$ and RTEL1$^{F/F}$ *POLE4*$^{-/-}$ infected or not with CRE (* $p< 0.05$).
