# Supplementary material for: Synthetic Lethality between DNA Polymerase Epsilon and RTEL1 in Metazoan DNA Replication
Source: Cell Rep. 2020 May 26;31(8):107675. doi: 10.1016/j.celrep.2020.107675 (PMC7262601; doi:10.1016/j.celrep.2020.107675)
Supplement: Document S2. Article plus Supplemental Information [file mmc2.pdf]

Cell Reports

Article

Synthetic Lethality between DNA Polymerase Epsilon and RTEL1 in Metazoan DNA Replication

Graphical Abstract

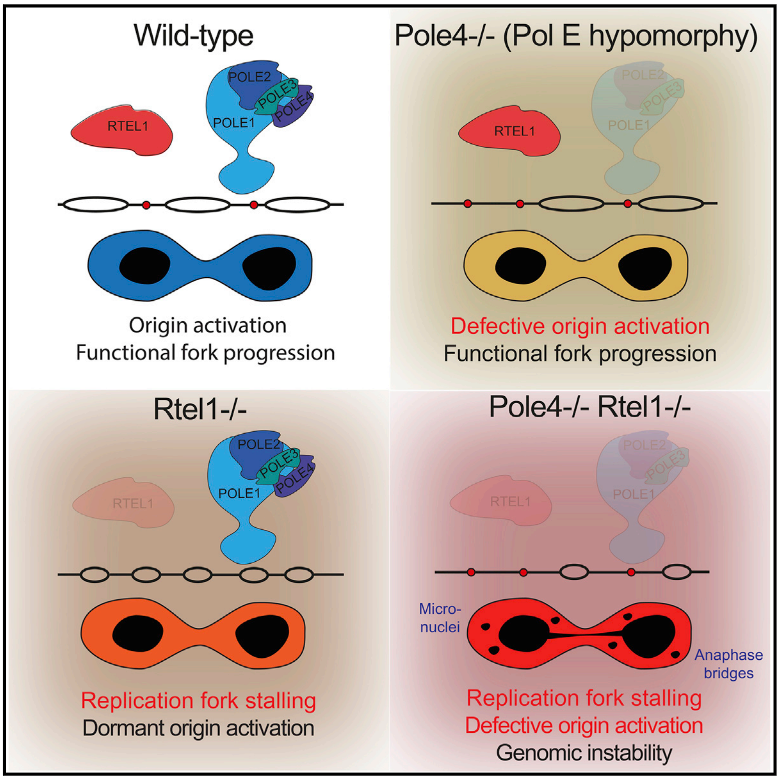

Highlights

- *rtel-1* is synthetic lethal with the loss of DNA polymerase epsilon in *C. elegans*
- *rtel-1; pole-4* double mutants accumulate Rad51 and RPA foci and fail to replicate
- Impaired DNA replication and genome instability in *Rtel1 Pole4* knockout mouse cells
- *Rtel1 Pole4* double knockout mouse cells exhibit fork asymmetry and defective origin activation

Authors

Roberto Bellelli, Jillian Youds, Valerie Borel, Jennifer Svendsen, Visnja Pavicic-Kaltenbrunner, Simon J. Boulton

Correspondence

simon.boulton@crick.ac.uk

In Brief

Bellelli et al. report that RTEL1 deficiency is synthetic lethal with the loss of *pole-4* in *C. elegans*/hypomorphy of Pol epsilon. An analysis of replication dynamics in *Rtel1*$^{-/-}$ *Pole4*$^{-/-}$ mouse cells revealed a combination of dysfunctional fork progression and defective origin activation, which cooperatively drive incomplete genomic replication and genetic instability.

Bellelli et al., 2020, Cell Reports *31*, 107675
May 26, 2020 © 2020 The Author(s).
https://doi.org/10.1016/j.celrep.2020.107675

CellPress

Cell Reports

CellPress
OPEN ACCESS

Article

Synthetic Lethality between DNA Polymerase Epsilon and RTEL1 in Metazoan DNA Replication

Roberto Bellelli,[1,2,3] Jillian Youds,[1,2] Valerie Borel,[1] Jennifer Svendsen,[1] Visnja Pavicic-Kaltenbrunner,[1] and Simon J. Boulton[1,4,*]

[1]The Francis Crick Institute, 1 Midland Road, NW1 1AT London, UK

[2]These authors contributed equally

[3]Present address: Center for Cancer Cell and Molecular Biology, Barts Cancer Institute, Queen Mary University of London, Charterhouse Square, Barbican, EC1M 6BE London, UK

[4]Lead Contact

*Correspondence: simon.boulton@crick.ac.uk

https://doi.org/10.1016/j.celrep.2020.107675

SUMMARY

Genome stability requires coordination of DNA replication origin activation and replication fork progression. RTEL1 is a regulator of homologous recombination (HR) implicated in meiotic cross-over control and DNA repair in *C. elegans*. Through a genome-wide synthetic lethal screen, we uncovered an essential genetic interaction between RTEL1 and DNA polymerase (Pol) epsilon. Loss of POLE4, an accessory subunit of Pol epsilon, has no overt phenotype in worms. In contrast, the combined loss of POLE-4 and RTEL-1 results in embryonic lethality, accumulation of HR intermediates, genome instability, and cessation of DNA replication. Similarly, loss of *Rtel1* in *Pole4*$^{-/-}$ mouse cells inhibits cellular proliferation, which is associated with persistent HR intermediates and incomplete DNA replication. We propose that RTEL1 facilitates genome-wide fork progression through its ability to metabolize DNA secondary structures that form during DNA replication. Loss of this function becomes incompatible with cell survival under conditions of reduced origin activation, such as Pol epsilon hypomorphy.

INTRODUCTION

DNA replication origins are established at thousands of sites throughout the genome through a combination of structural and functional chromatin determinants that promote loading of inactive MCM2-7 double hexamers around DNA replication origins (Fragkos et al., 2015). At the G1-S transition, DDK- and CDK-dependent phosphorylation events drive the formation and activation of the CMG (CDC45-MCM2-7-GINS), the eukaryotic replicative helicase, followed by establishment of two symmetric replication forks that initiate DNA synthesis (Bell and Labib, 2016). The bulk of DNA replication at active replication forks is performed by the conserved DNA polymerase complexes Pol delta and Pol epsilon, which act on the lagging and leading strand, respectively (Burgers and Kunkel, 2017). The latter is also considered to be a stable component of the replisome and is required for efficient CMG formation in budding yeast (Bell and Labib, 2016). DNA replication in metazoans also requires the function of several helicases and replisome-associated factors to prevent inappropriate transactions at the replication fork and to avert persistent fork stalling events (León-Ortiz et al., 2014; Dungrawala et al., 2015).

The helicase RTEL1 was identified as the first metazoan anti-recombinase, which facilitates DNA repair and regulates cross over formation in meiosis in *C. elegans* (Barber et al., 2008; Youds et al., 2010). *rtel-1* mutant worms display reduced brood size and viability, sensitivity to DNA damaging agents, and elevated meiotic recombination. Biochemical studies show that RTEL1 can efficiently disassemble D-loop recombination intermediates, suggesting that RTEL-1 might disassemble these intermediates to promote non-crossover repair, likely through synthesis-dependent strand annealing. Genetic analysis also revealed that *rtel-1* is synthetic lethal when combined with mutations in *dog-1/FANCJ*, *mus-81*, *him-6/BLM*, and *rcq-5*, all of which are homologs of genes involved in human genetic diseases and the maintenance of genome stability at replication forks (Barber et al., 2008). Indeed, mutants of these genes when combined with *rtel-1* displayed persistent RAD-51 foci in the germline and embryonic lethality, indicating that RTEL-1 is essential in their absence. However, where and when RTEL1 activity is essential in nematodes remained to be established. Subsequent studies showed that RTEL1 facilitates efficient telomere and genome-wide replication in vertebrates (Vannier et al., 2013), but its precise function during DNA replication remains unclear.

To gain an improved understanding of the role of RTEL-1 in maintaining genome stability, we conducted a genome-wide RNAi screen to identify genes that, when knocked down with RNAi in the *rtel-1* mutant background, cause synthetic lethality, but not in the wild-type. This genetic screen identified multiple genes involved in DNA replication, such as TOPBP1, GINS complex subunits PSF2 and PSF3, RFC-1, FEN-1, and CDT1, as well

This is an open access article under the CC BY license (http://creativecommons.org/licenses/by/4.0/).

as three components of DNA Pol epsilon, which we chose to further investigate. Strains lacking the non-essential subunit of Pol epsilon, *pole-4*, exhibit no overt phenotype under normal or DNA damaging conditions. Strikingly, however, the *pole-4; rtel-1* double mutant is 100% synthetic embryonic lethal and presents with persistent homologous recombination (HR) intermediates, extensive genome instability, and cessation of DNA replication. We proceed to show that this synthetic lethal interaction is conserved in mammalian cells. A combined loss of RTEL1 and POLE4 in primary mouse cells also inhibits cellular proliferation and results in extensive genetic instability. Molecular analysis of DNA replication dynamics in *Rtel1-Pole4* double knockout cells revealed a combination of dysfunctional fork progression and origin activation, which leads to fork stalling and genome under-replication.

Our data, although pointing to conserved functions in metazoans for RTEL1 in replication fork progression and POLE4 in maintaining Pol epsilon complex stability, reveal an un-appreciated interplay between replication origin activation and fork progression required for genome-wide DNA replication and the maintenance of genome stability.

RESULTS

rtel-1 Is Synthetic Lethal with Members of the DNA Polymerase Epsilon Complex in *C. elegans*

RTEL1 is dispensable for viability in the nematode *C. elegans*, an organism amenable to genetic manipulation and a potent system to identify synthetic interactions *in vivo*. To further interrogate the functions of RTEL1 in genome stability in *C. elegans*, we performed a genome-wide RNAi screening in N2(wild type) and *rtel-1* mutant worms (Kamath et al., 2003) by using a library of 16,256 genes (Figure S1A). Following secondary screens to confirm our initial hits, we identified a number of genes with established roles in DNA replication whose RNAi caused lethality in the *rtel-1* mutant but not in an N2(wild-type) strain (Figure S1B). These genes included *rfc-1/RFC1*, *mus-101/TOPBP1*, *crn-1/FEN1*, *F31C3.5/PSF2*, *Y65B4BR.8/PSF3*, and *cdt-1/CDT1*. Interestingly, we also identified three components of the DNA Pol epsilon complex: *F33H2.5* (*pole-1*), *F08B4.5* (*pole-2*), and *T26A5.8* (*pole-3*) (Figure 1A). A fourth component of the complex, *Y53F4B.3* (*pole-4*), was not present in the RNAi library. In secondary screens, all three DNA Pol epsilon components knocked down by RNAi showed dramatic synthetic lethal phenotypes in the *rtel-1* mutant (Figure S1B), which we decided to explore further (Figure 1A).

We observed that RNAi knockdown of *pole-1*, *pole-2*, or *pole-3* caused a significant reduction in brood size and viability in *rtel-1* mutants compared with N2(wild-type) worms (Figure 1B). Orthologs of *pole-1* and *pole-2* are essential for viability in budding yeast; thus, RNAi in worms likely produced a partial knockdown of these genes. Furthermore, variability in knockdown efficiency could account for the different levels of synthetic lethality between *rtel-1*, *pole-1*, and *pole-2*. Importantly, we did not observe any overt synthetic lethality when we knocked down the major subunit of DNA Pol delta, *F12F6.7*, in the *rtel-1* background, suggesting that the genetic interaction between *rtel-1* and DNA Pol epsilon does not extend to DNA Pol delta (Figure S1C). *pole-3* is predicted to be a non-essential component of the complex, and therefore, RNAi of this gene had a less dramatic effect on *rtel-1* mutants than *pole-1* or *pole-2* RNAi (Figure 1B). We proceeded to examine the germlines of these animals for defects that could explain the source of the observed synthetic lethality. After RNAi for *pole-1* or *pole-2*, *rtel-1* mutants exhibited increased levels of RAD-51 foci in the mitotic zone of the germline, which is the only region of active DNA replication in the *C. elegans* germline. In contrast, RAD-51 foci were not observed in N2(wild-type) animals fed with the same RNAi, suggesting that replication defects might underlie the lethality in *rtel-1; pole-1*(RNAi) and *rtel-1; pole-2*(RNAi) animals (Figure 1C).

rtel-1 Is Synthetic Lethal with *pole-4* in *C. elegans*

To confirm our observations with RNAi, we obtained a genetic deletion of *pole-4*, *tm4613*, which removed the majority of the coding region of the gene, apart from the first 35 nucleotides of exon 1; this likely represents a bona fide null allele (Figure S2A). *pole-4(tm4613)* mutants appeared superficially wild type, and the loss of *pole-4* did not result in any significant loss of viability. *rtel-1* mutants show greater than 90% viability as previously described (Barber et al., 2008). In contrast, *rtel-1; pole-4* double mutant animals displayed a dramatic synthetic embryonic lethal phenotype (Figure 2A), wherein none of the progeny were viable. This was associated with a dramatic reduction in DNA replication, as observed by attenuated incorporation of Cy3-dUTP in double mutant worms (Figure 2B).

Because DNA Pol epsilon has been implicated in activation of the intra-S-phase checkpoint, we questioned whether the absence of *pole-4* might also inactivate the replication checkpoint in worms (Navas et al., 1995). However, in contrast to this hypothesis, DAPI staining of the germlines of *rtel-1; pole-4* double mutants showed enlarged mitotic nuclei and fewer nuclei in the mitotic zone than wild-type animals or either single mutant, a phenotype associated with mitotic replication arrest due to checkpoint activation. To quantify the mitotic arrest, we counted the number of nuclei in a single plane of focus within 45 μm of the distal tip cell of the mitotic zone. *rtel-1; pole-4* double mutants had fewer mitotic nuclei than N2(wild-type) or either single mutant alone (Figure 2C). Thus, the number of nuclei in *rtel-1; pole-4* animals was more similar to wild-type animals treated with 40 μM hydroxyurea (HU) than either single mutant, indicating that replication stress and activation of the DNA replication checkpoint were indeed present in *rtel-1; pole-4* double mutants. We conclude that the absence of *pole-4* does not compromise the replication checkpoint in worms. This is in accordance with recent findings in both *Pole4*$^{-/-}$ mouse embryo fibroblasts and CRISPR knockout human cells (Bellelli et al., 2018; Hustedt et al., 2019).

Finally, we examined the mitotic regions to determine the types of DNA damage occurring spontaneously in *rtel-1; pole-4* double mutants. Staining with an anti-RPA antibody showed an accumulation of RPA foci in *rtel-1; pole-4* animals, but not in single mutants, which is indicative of replication stress and DNA single-strand accumulation in the double mutant, potentially due to uncoupling between DNA polymerases and the CMG helicase (Figures 2D and S2B).

CellPress
OPEN ACCESS

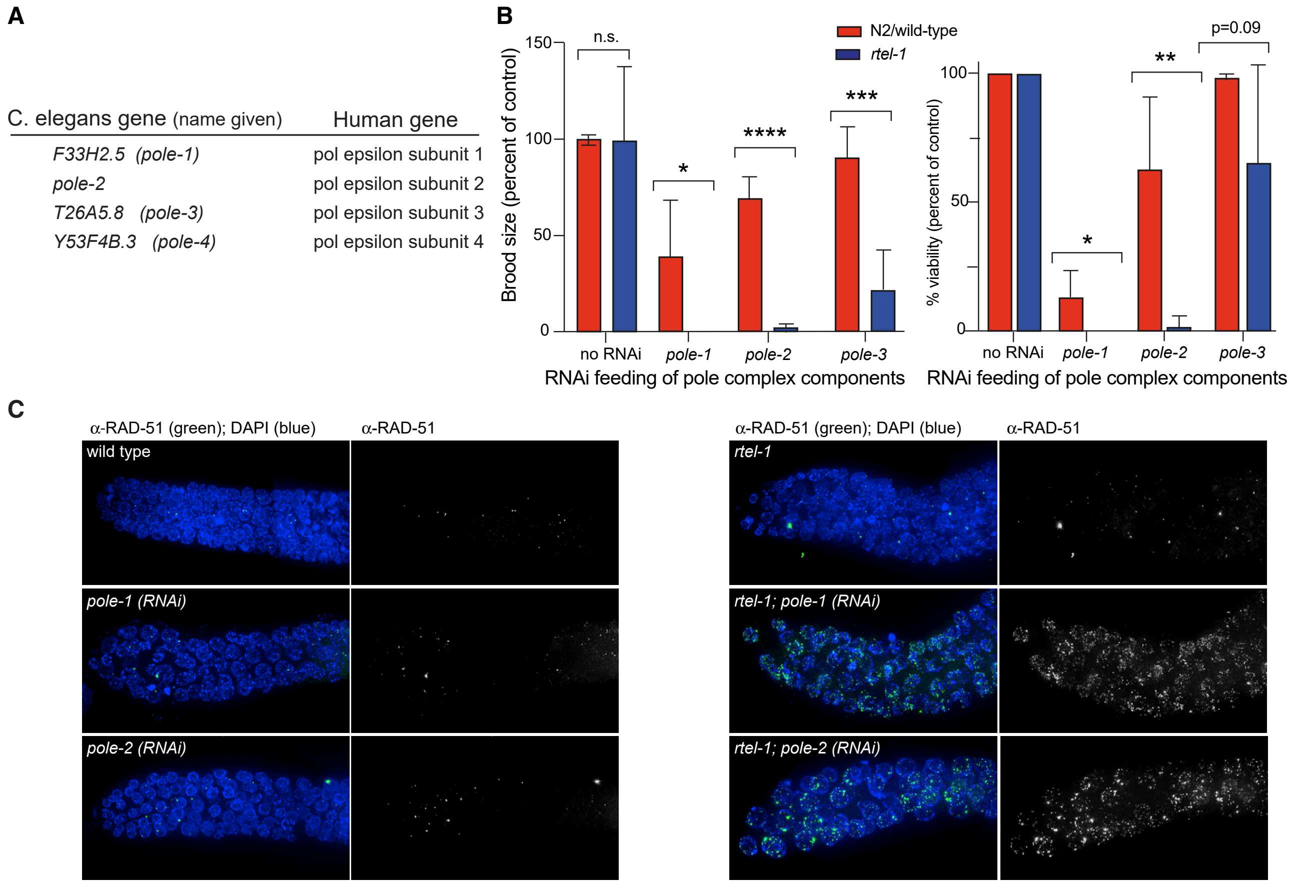

Figure 1. Knockdown of Polymerase Epsilon Components by RNAi Causes Synthetic Lethality in *rtel-1* Mutant Background

(A) *C. elegans* gene names of the four polymerase epsilon components and their corresponding human homologs.

(B) Total brood size and percent viability after feeding either no RNAi or RNAi for *pole-1*, *pole-2*, or *pole-3* in the N2(wild-type) or *rtel-1* mutant. Brood size and percent viability are both normalized based on untreated N2(wild-type) or *rtel-1* control animals. (*p < 0.05, *p < 0.01, ***p < 0.001, ****p < 0.0001; n.s., not significant).

(C) RAD-51 staining of mitotic zones of N2(wild-type) or *rtel-1* animals fed either with no RNAi, *pole-1*, or *pole-2* RNAi.

Images are composites of several images stitched together. Error bars in all graphs represent standard deviation (SD) of the mean.

Mus81- and *Rfs1*-Dependent Processing of Replication Intermediates in *rtel-1*; *pole4* Worms

In eukaryotes, persistent fork stalling events are associated with recruitment of the RAD-51 recombinase to protect newly synthesized DNA from nucleolytic degradation and promote recombination-dependent fork restart and/or processing into double-strand breaks (DSBs) for canonical DNA repair (Bhat and Cortez, 2018). Thus, we stained mutant strains with RAD-51 antibodies to monitor the presence and resolution of HR intermediates. Strikingly, although mitotic RAD-51 foci were rarely observed in N2(wild-type) or single mutant animals, *rtel-1; pole-4* double mutants showed a strong accumulation of RAD51 foci in the mitotic zone (Figure 3A). *rtel-1; pole-4* animals also displayed greater numbers of RAD-51 foci throughout the meiotic regions of the germline, and these foci persisted through late pachytene when meiotic DSB repair is normally complete (Figure S3A). This data suggest that DNA damage occurring spontaneously during DNA replication in *rtel-1; pole-4* double mutants persists into meiosis.

To analyze the consequences of replicative damage accumulation in *rtel-1; pole-4* double mutants, we analyzed diakinesis nuclei. In N2(wild-type) animals, six DAPI-stained bodies are present at diakinesis, which correspond to paired homologous chromosomes held together by a single chiasmata. As expected, N2(wild-type) animals, as well as *rtel-1* and *pole-4* single mutants, presented with six intact DAPI-stained bodies. In contrast, *rtel-1; pole-4* animals displayed a wide range of chromosomal defects at diakinesis, ranging from chromosome fragments to unpaired sister chromatids, chromosome fusions, and constrictions on the chromosome arms (Figures 4B and S3B). Quantification of the number of DAPI-stained bodies at diakinesis showed that 41% of *rtel-1; pole-4* diakinesis nuclei had greater than six bodies. However, many of those that showed a correct number of DAPI-stained bodies displayed constrictions on the arms of chromosomes, suggesting the presence of significant chromosome damage. Thus, we conclude that DNA damage arising during DNA replication in the absence of *rte-l1* and *pole-4* persists as nuclei progress through meiosis, resulting in

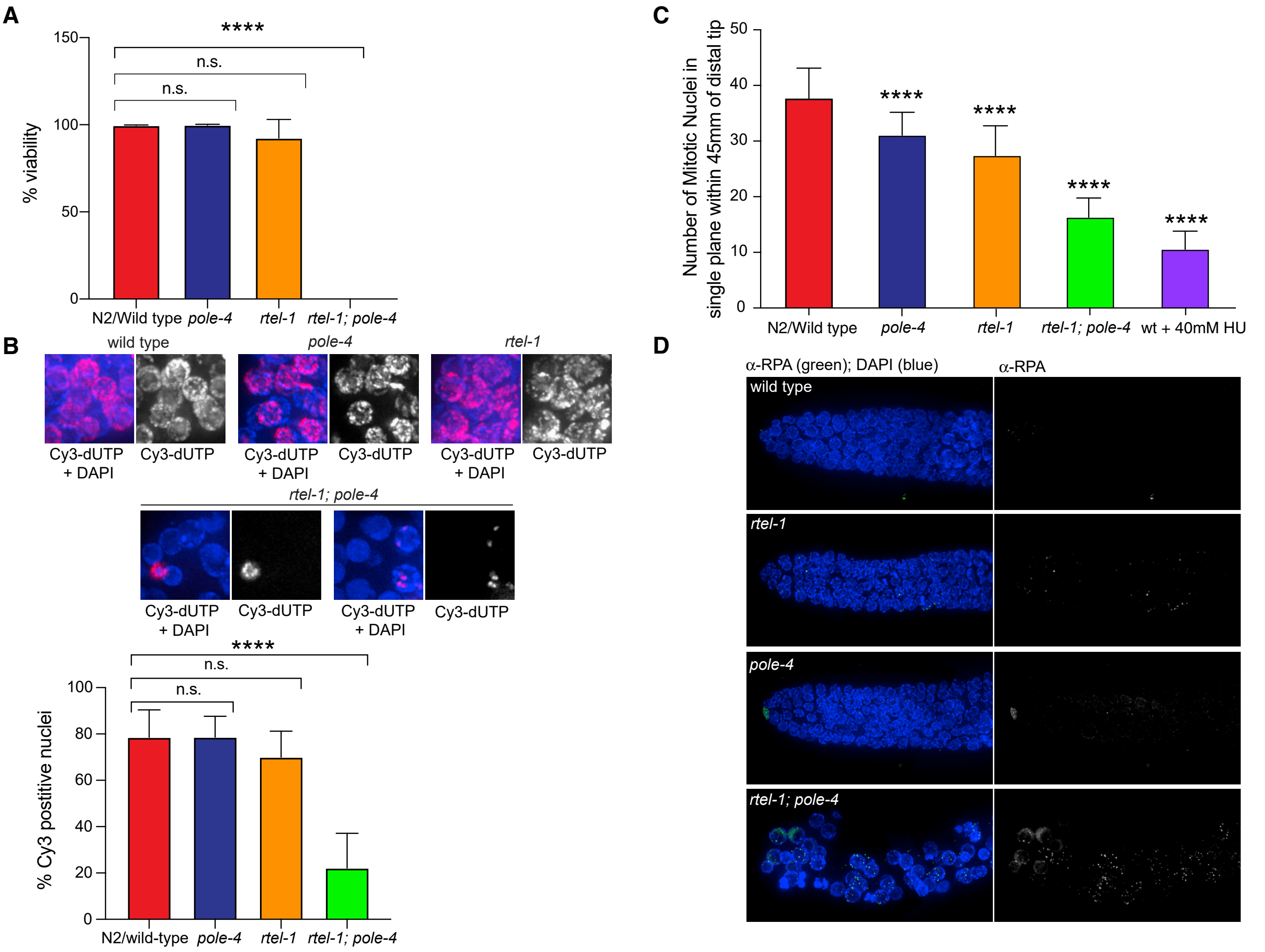

Figure 2. *rtel-1* Is Synthetic Lethal in Combination with *pole-4* Deletion

(A) Progeny viability in N2(wild-type), *pole-4*, and *rtel-1* single mutants and *rtel-1; pole-4* double mutants (****p < 0.0001; n.s., not significant).

(B) Top: representative images of Cy3-dUTP incorporation in mitotic nuclei in N2(wild-type), *pole-4*, and *rtel-1* single mutants and *rtel-1; pole-4* double mutant worms. Bottom: bar graphs showing the percentage of Cy3-dUTP-positive cells in the described genetic backgrounds (****p < 0.0001; n.s., not significant).

(C) Average number of mitotic nuclei counted in a single plane of focus within 45 μm of the distal tip cell as a measure of mitotic arrest and checkpoint activity in the genotypes shown. N2(wild-type) animals treated for 24 h with 40 mM hydroxyurea (HU) were used as a control (****p < 0.0001).

(D) RPA staining of mitotic zones in the indicated genotypes (see also Figure S2B for quantification).

Images are composites of several images stitched together. Error bars in all graphs represent standard deviation (SD) of the mean.

meiotic chromosomal defects and the subsequent lethality of *rtel-1; pole-4* embryos.

In our previous work, we identified a synthetic lethal genetic interaction between *rtel-1* and *dog-1*, the FANCJ homolog in *C. elegans* (Barber et al., 2008; Youds et al., 2008). DOG-1 is involved in inter-strand cross-link repair and is also responsible for maintaining the stability of tracts of poly-guanine DNA in metazoans (Cheung et al., 2002). DOG-1 is believed to unwind secondary structures that may form in these G-rich sequences during replication. Thus, in its absence, deletions occur in tracts of poly-guanine greater than 18 nucleotides in length.

We noted that similar to *rtel-1; pole-4* animals, *rtel-1; dog-1* double mutants display mitotic RAD-51 foci, suggesting a possible link between the defects observed in these two strains. With this in mind, we tested whether or not *pole-4* might also be synthetic lethal with *dog-1*. However, *dog-1; pole-4* double mutants showed greater than 90% viability (Table S1). Furthermore, no G-tract deletions were observed in *pole-4* or *rtel-1* single mutants or in *rtel-1; pole-4* double mutants. The frequency of G-tract deletions in the *dog-1* background was also unchanged after feeding with *pole-2*(RNAi) (Table S2), indicating that the lethality in *rtel-1; pole-4* is unrelated to poly G-tract instability.

To understand the nature of the DNA damage that accumulates in *rtel-1; pole-4* mutant animals, we analyzed the dependence of mitotic RAD-51 foci formation on MUS-81 and RFS-1. MUS-81 is a structure-specific endonuclease that has been implicated in processing DNA damage intermediates at the replication forks

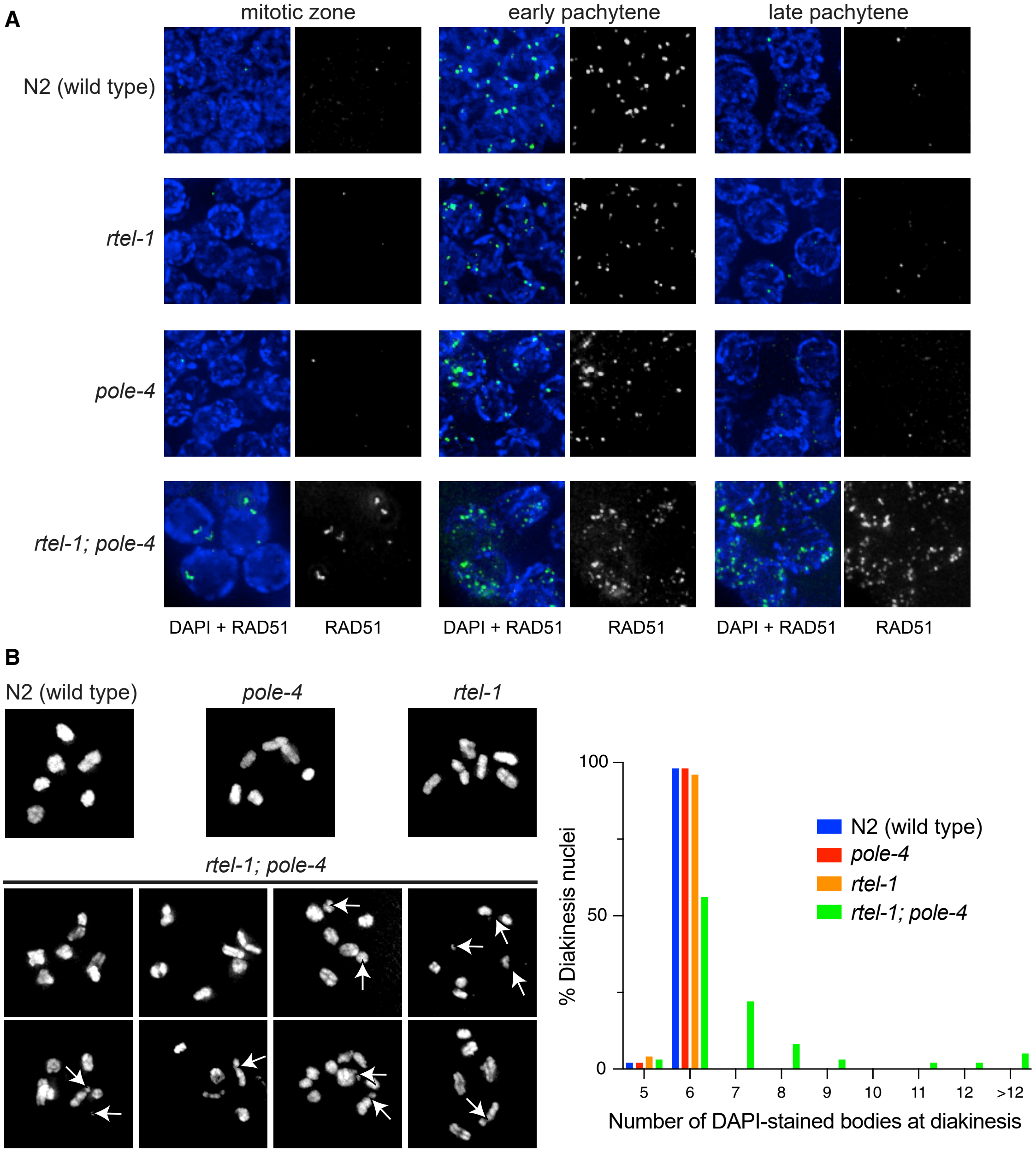

Figure 3. RAD-51 Foci and Chromosome Aberrations Are Elevated in *rtel-1; pole-4* Double Mutants

(A) Representative images of RAD-51 foci in mitotic zones and early pachytene and late pachytene in the indicated genotypes. Greyscale images show RAD-51 only.

(B) Left: top panels show representative images of N2(wild-type), *pole-4*, and *rtel-1* single mutant diakinesis nuclei showing six DAPI-stained bivalents present; bottom panels show examples of the chromosome defects observed in *rtel-1; pole-4*, including chromosome fragments, constrictions on chromosome arms, chromosome fusions, and unpaired sister chromatids. Right: quantification of the number of DAPI-stained bodies present at diakinesis in each of the indicated strains (see also Figure S3B).

(Dehé and Gaillard, 2017), whereas RFS-1 is a RAD-51 paralog that is required for RAD-51 loading specifically at stalled replication forks, but not at sites of fork collapse or DNA DSBs (Ward et al., 2007). If the RAD-51 foci in *rtel-1; pole-4* are dependent on RFS-1, this would suggest that damage sites represent stalled/blocked replication forks rather than collapsed forks or breaks. To this end, we constructed two balanced strains: *rtel-1 mus-81*/hT2[gfp]; *pole-4* and *rtel-1*/hT2[gfp]; *pole-4; rfs-1*/hT2[gfp] from which we could isolate homozygous triple mutants and stain for the presence of RAD-51. Compared to *rtel-1; pole-4* double mutants, both *rtel-1; mus-81; pole-4* and *rtel-1; pole-4; rfs-1* triple mutant animals exhibited statistically fewer

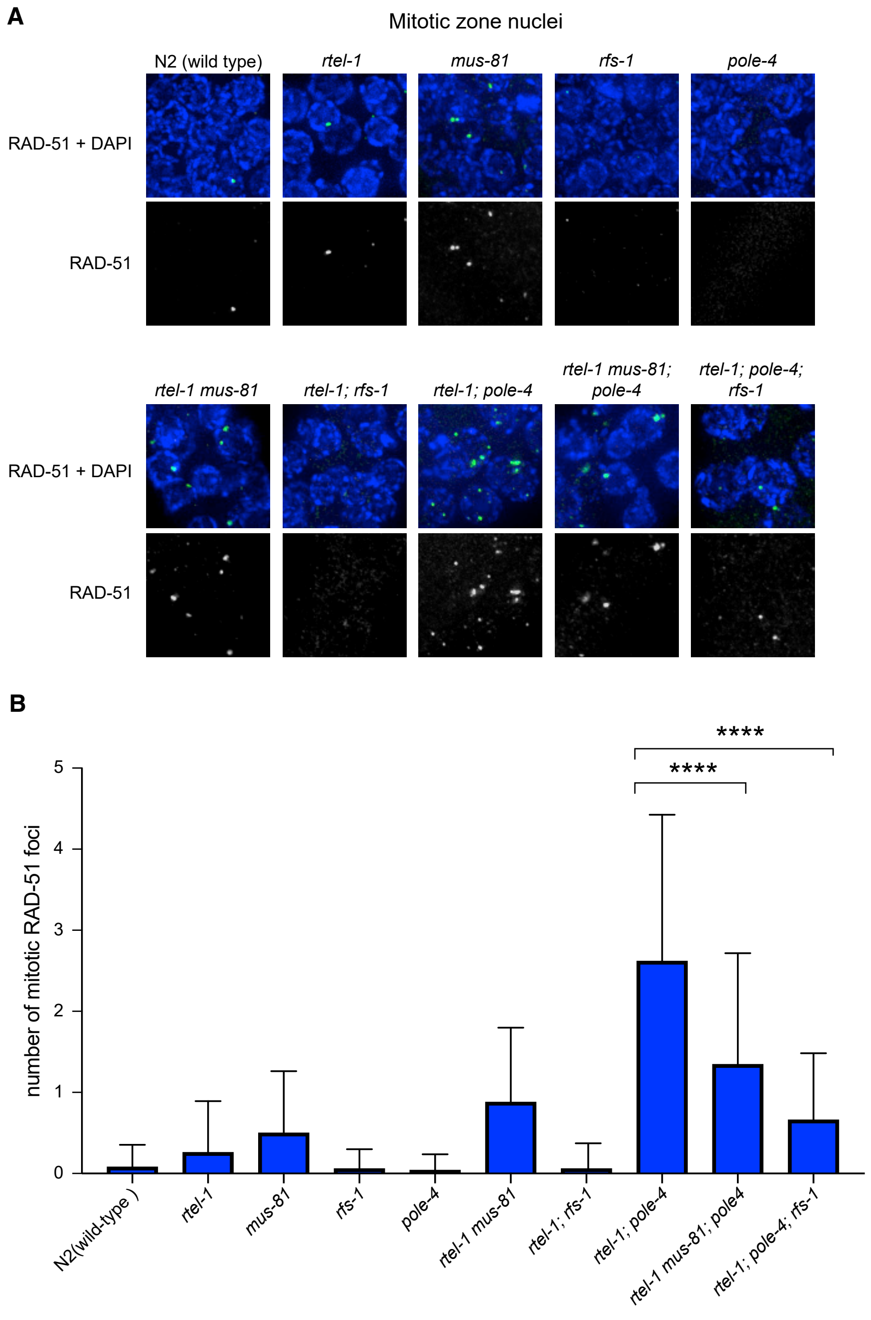

Figure 4. Mitotic RAD-51 Foci in *rtel-1; pole-4* Double Mutants Are Dependent on Both MUS-81 and RFS-1

(A) Representative images of mitotic nuclei stained with anti-RAD-51 in the indicated genotypes.

(B) Quantification of the average number of RAD-51 foci per mitotic nucleus in the described genotypes is shown (****$p < 0.0001$). Error bars represent standard deviation (SD) of the mean.

mitotic RAD-51 foci (Figures 4A and 4B), indicating that in *rtel-1; pole-4* mutants, RAD-51 foci formation is partially dependent on both MUS-81 and RFS-1. Overall, RAD-51 foci were more significantly reduced in *rtel-1; pole-4; rfs-1* than in *rtel-1; mus-81; pole-4*, suggesting a greater dependence on RFS-1 than on MUS-81. Given that MUS-81 and HIM-9/XPF-1 exhibit redundancy in generating recombination substrates at inter-strand cross-links (Ward et al., 2007), we speculate that both MUS-81 and HIM-9/XPF-1 might independently process replication intermediates present in *rtel-1; pole-4* animals, explaining the partial dependency on MUS-81 for RAD-51 foci formation. Despite the overall reduction in the number of RAD-51 foci, we did not observe a rescue of viability in *pole-4; rtel-1; rfs-1* and *rtel-1; mus-81; pole-4* triple mutant animals, thus suggesting that RAD-51-dependent HR events might promote cell survival upon persistent replication fork stalling in *pole-4; rtel-1* mutant worms (Table S1).

Proliferative Failure and Impaired DNA Replication in *Rtel1-Pole4* Double Knockout Cells

We recently reported the generation of a *Pole4* knockout mouse, which presents with intra- and extra-uterine growth restriction, developmental abnormalities, and lymphopenia. *In vitro*, *Pole4*$^{-/-}$ mouse embryonic fibroblasts (MEFs) exhibit Pol epsilon complex instability and spontaneous DNA damage accumulation, which we attributed to reduced origin activation and replication stress (Bellelli et al., 2018). To investigate the consequences of the loss of *RTEL1* in *POLE4*$^{+/+}$ and *POLE4*$^{-/-}$ MEFs, we infected conditional *Rtel1*$^{F/F}$ *Pole4*$^{+/+}$ and *Rtel1*$^{F/F}$ *Pole4*$^{-/-}$ primary MEFs with adenovirus expressing GFP-CRE or empty GFP. Transduction of *Rtel1*$^{F/F}$ MEFs resulted in the expected loss of the floxed RTEL1 alleles and elimination of endogenous RTEL1 protein within 72 h (Figure S4). Strikingly, the loss of both *Rtel1* and *Pole4* resulted in a complete block of cellular proliferation in double knockout cells, as assessed by cumulative population doublings analysis (Figure 5A). Of note, both *Pole4*$^{-/-}$ and *Rtel1*$^{F/F}$ cells showed a variable degree of reduced cellular proliferation, which complicate the identification of a specific synthetic interaction. In addition to this, the concomitant loss of *Pole4* and *Rtel1* was associated with an overall reduction in EdU incorporation, similarly to that observed in *rtel-1; pole4* mutant worms, suggesting a cooperative function for Pol epsilon and RTEL1 in promoting genome-wide replication in both worms and mammalian cells in culture (Figures 5B and 5C).

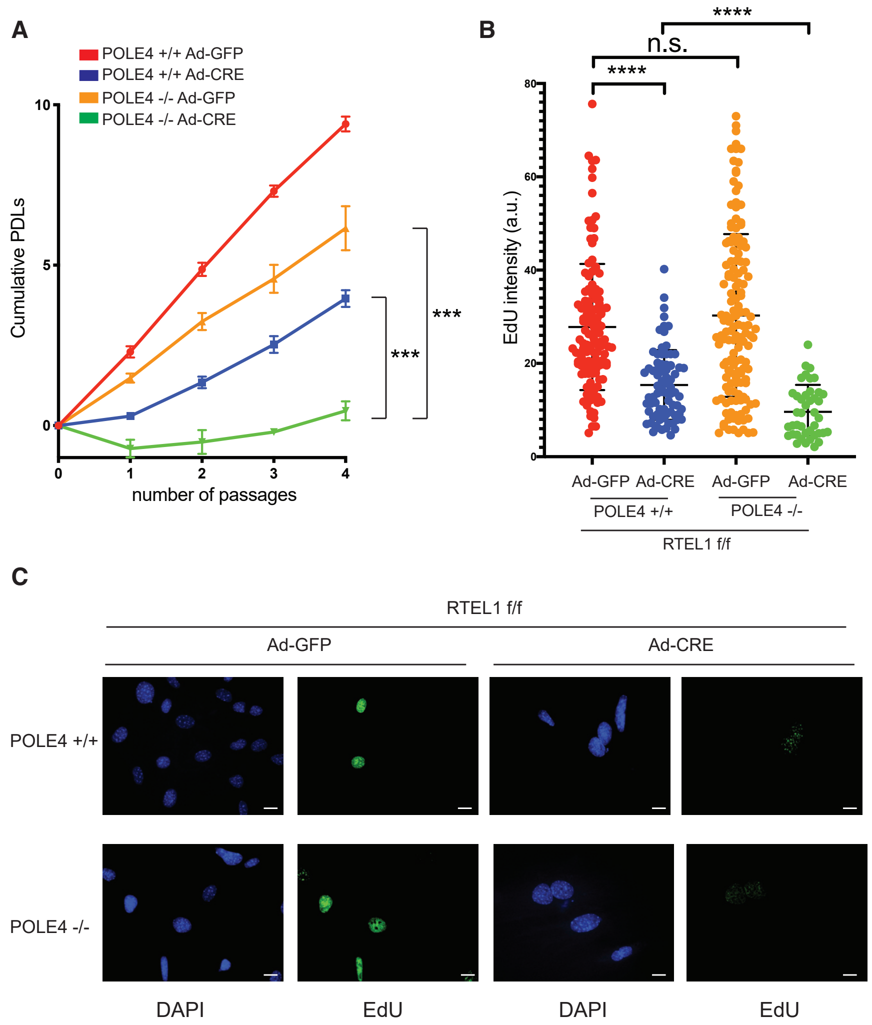

Figure 5. Reduced Growth and Impaired EdU Incorporation in *RTEL1*$^{F/F}$ *POLE4*$^{-/-}$ Primary Mouse Embryo Fibroblasts (MEFs)

(A) Cumulative population doublings (PDLs) of *RTEL1*$^{F/F}$ *POLE4*$^{+/+}$ and *RTEL1*$^{F/F}$ *POLE4*$^{-/-}$ MEFs infected with adenovirus expressing GFP-CRE or empty GFP. Cells were seeded for PDL analysis 72 h after infection and cultured according to a standard 3T3 protocol. Bars represent mean ± SD of triplicate experiments (***$p < 0.001$).

(B) Bar graphs showing EdU intensity staining (arbitrary units) of *RTEL1*$^{F/F}$ *POLE4*$^{+/+}$ and *RTEL1*$^{F/F}$ *POLE4*$^{-/-}$ infected or not with CRE. Cells were analyzed for EdU incorporation 72h after infection with GFP-CRE or empty GFP. Bars represent mean ± SD of triplicate experiments (****$p < 0.0001$; n.s., not significant).

(C) Representative images of EdU staining from *RTEL1*$^{F/F}$ *POLE4*$^{+/+}$ and *RTEL1*$^{F/F}$ *POLE4*$^{-/-}$ MEFs infected or not with CRE recombinase. Scale bars, 16 μm.

Extensive DNA Damage and Chromosomal Instability upon Combined Loss of *Rtel1* and *Pole4*

To further characterize the mechanism responsible for reduced growth and EdU incorporation in *Rtel1*-*Pole4* double knockout mouse cells, we analyzed by immunofluorescence the presence of markers of DNA damage, including γH2AX and 53BP1 foci, in *Rtel1*$^{F/F}$ *POLE4*$^{+/+}$ and $^{-/-}$ MEFs infected or not with CRE (Panier and Boulton, 2014). In accordance with a failure to complete DNA replication, the absence of both RTEL1 and POLE4 lead to a strong increase in both γH2AX and 53BP1 foci-positive cells (Figures 6A and 6B). More importantly, the combined loss of POLE4 and RTEL1 was associated with a striking increase in nuclear blebbing and micronuclei formation, suggestive of extensive chromosomal instability (Figure 6C). In addition to this, atypical nuclear structures and mitotic bridges were observed that are suggestive of incomplete DNA replication (Figures S5A, S5B, and S5C).

Loss of RTEL1 and POLE4 Leads to Replication Stress and Reduced Fork Extension Rates

We previously showed that RTEL1 is involved in telomere and genome-wide replication (Vannier et al., 2013). Conditional deletion of *Rtel1* in MEFs leads to reduced replication fork extension rates, reduced inter-origin distance, and increased fork asymmetry, which are all suggestive of fork stalling and increased origin use due to dormant origin activation (Ge et al., 2007; Ibarra et al., 2008; Vannier et al., 2013). Conversely, *Pole4*$^{-/-}$ cells exhibit increased inter-origin distances and enhanced fork speed associated with heightened replication stress and increased fork asymmetry (Bellelli et al., 2018).

To understand the consequences of the combined loss of RTEL1 and POLE4 on replication fork activation and elongation, we analyzed the replication dynamics of single and double mutant cells. To this aim, we infected *RTEL1*$^{F/F}$ *POLE4*$^{+/+}$ or *RTEL1*$^{F/F}$ *POLE4*$^{-/-}$ primary MEFs with adenovirus expressing GFP-CRE or empty GFP and, 72 h after CRE-mediated excision of RTEL1, pulse-labeled cells with CldU and IdU and performed DNA fiber analysis, as previously described (Figure 7A; Bellelli et al., 2018). Consistent with previous studies, the loss of RTEL1 led to a reduction in fork speed, increased fork asymmetry, and reduced inter-origin distance, whereas the loss of POLE4 led to a significant increase in fork extension rates due to reduced origin activation and increased inter-origin distance and fork asymmetry (Figures 7B, 7C, and 7D)

Strikingly, when we analyzed fork extension rates in *Rtel1*-*Pole4* double knockout cells, we observed a reduction in fork speed compared with both wild-type and *Rtel1*-null-only cells, suggestive of compromised fork elongation upon concomitant loss of RTEL1 and POLE4. In agreement with this hypothesis, more than 70% of newly activated replication origins featured asymmetry of newly incorporated nucleotide tracks (Figures 7B and 7C). However, distinct from that observed in *Rtel1*-null-only cells, double mutant cells did not exhibit a significant

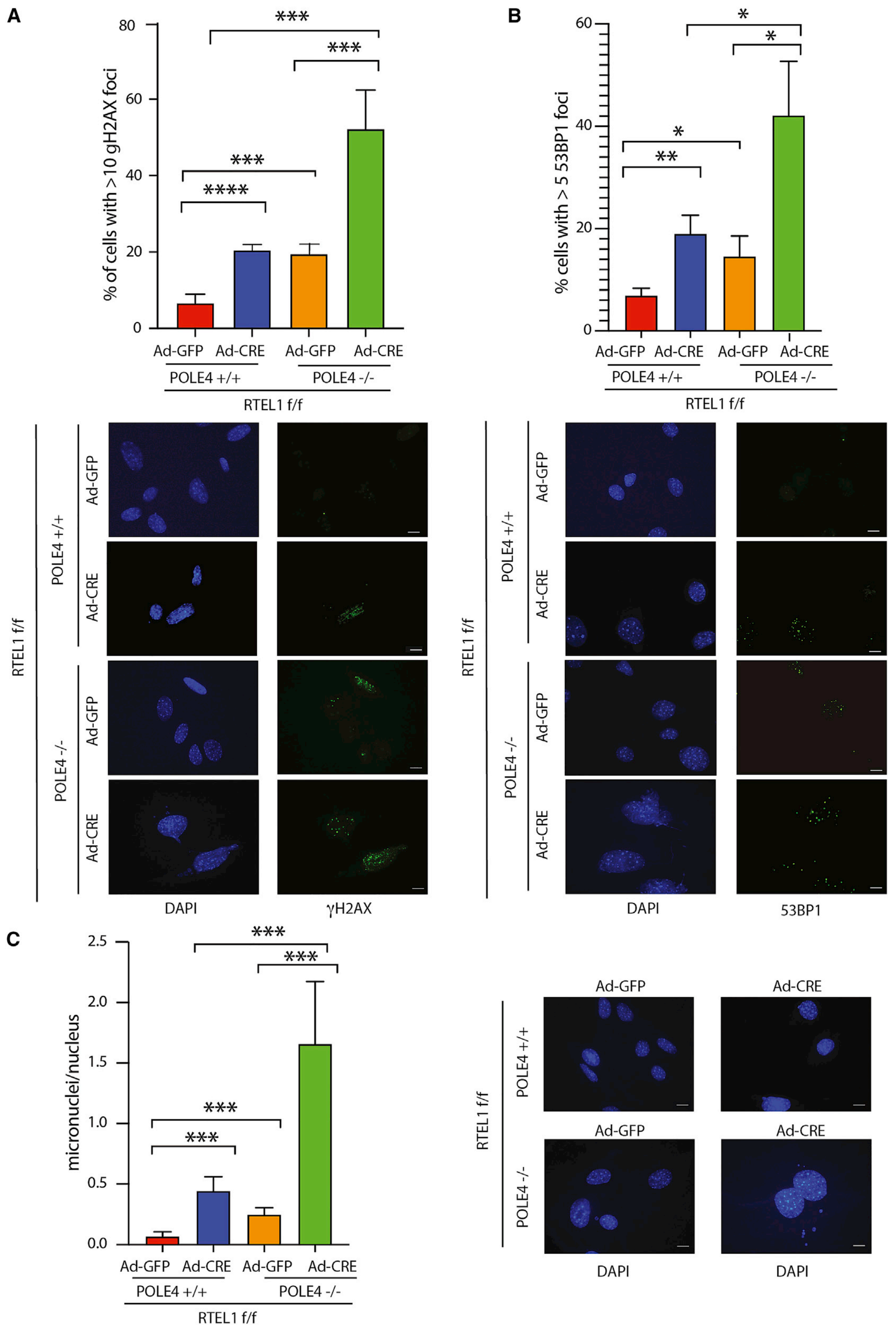

(legend on next page)

CellPress
OPEN ACCESS

reduction in inter-origin distance, which is suggestive of a failure to efficiently activate dormant replication origins (Figure 7D).

DISCUSSION

Here, we uncover a synthetic lethal interaction between the RTEL1 helicase and DNA Pol epsilon in the nematode *C. elegans*. RNAi-mediated loss of Pol epsilon complex subunits *pole-1*, *pole-2*, and *pole-3* conferred reduced viability in *rtel-1* mutant worms associated with elevated replication stress, as shown by RPA and RAD-51 foci accumulation, in both mitotic and meiotic zones. The viability of a strain lacking the fourth subunit of Pol Epsilon, *pole-4*, allowed us to genetically combine the loss of *rtel-1* and *pole-4* in *C. elegans*, which revealed a complete loss of viability upon removal of both RTEL-1 and POLE-4 in nematodes. We establish that this synthetic lethal interaction is also conserved in vertebrates and that, in both worms and mouse cells, the combined loss of Rtel1 and Pole4 confers extensive genome instability and cessation of DNA replication.

Importantly, a synthetic lethal interaction was also observed between *rtel-1* and other DNA replication genes required for the initiation of DNA replication, such as Topbp1 and psf2-psf3, which are components of the GINS complex. Topbp1 is the homolog of budding yeast Dpb11, which binds to phosphorylated Sld2 and Sld3 upon CDK activation and promotes pre-loading complex formation by recruiting the GINS complex in concert with Pol epsilon (Muramatsu et al., 2010). The fact that Dpb11, Psf2-Psf3 (in the context of GINS), and Pol epsilon directly bind to each other and are required for a specific step of DNA replication initiation strongly points to a direct and mechanistic connection between RTEL1 and replication fork progression in nematodes. Consistent with this hypothesis, we did not observe any evidence of a synthetic lethal interaction between RTEL1 and DNA Pol delta.

In agreement with a profound replication defect, RAD-51 foci accumulate in *rtel-1; pole-4* mutants and were partially dependent on MUS-81, a structure-specific endonuclease previously reported to process stalled replication intermediates to promote HR-dependent rescue of collapsed replication forks (Dehé and Gaillard, 2017). RAD-51 foci were also dependent on the Rad51 paralog RFS-1, which we previously showed to be exclusively required for RAD-51 loading at stalled replication forks (Ward et al., 2007).

Similar to the observations in worms, the combined loss of *Pole4* and *Rtel1* in MEFs led to a block to cellular proliferation, which was associated with DNA damage, genome instability, and incomplete DNA replication. At the molecular level, the loss of RTEL1 in a *Pole4* knockout background led to a substantial increase in replication fork asymmetry with reduced replication fork extension rates, which are all hallmarks of replication stress (Zeman and Cimprich, 2014; Técher et al., 2017). However, in contrast to that observed in a *Pole4* wild-type background, where the loss of *Rtel1* leads to reduced inter-origin distance due to dormant origin activation, double knockout cells failed to efficiently activate dormant origins.

We previously showed that RTEL1 binds to the proliferating cell nuclear antigen (PCNA) by its PIP-box domain and that this function is required for replication, potentially though problematic sequences, such as G4-DNA structures (Vannier et al., 2013). However, the lack of synthetic lethality between *pole-4* and *dog1/FANCJ*, which is required for G4-DNA stability in worms (Cheung et al., 2002; Youds et al., 2008), likely excludes that G4-DNA is a source of synthetic lethality in *rtel-1; pole-4* double mutants. In addition to this, it was recently shown that RTEL1 is required for the bypass of DNA-protein cross-links as well as non-covalent nucleoprotein complexes (Sparks et al., 2019). This function of RTEL1 appears to be PIP-box independent and suggests additional mechanisms of RTEL1 recruitment at the replication fork. If and how this newly described function of RTEL1 is required for genome-wide replication fork progression remains to be addressed.

In summary, through the identification of a novel genetic interaction between RTEL1 and Pol epsilon in nematodes, we reveal an essential role for RTEL1 in DNA replication under conditions of reduced origin firing and unveil a fundamental requirement for the strict coordination between origin activation and fork elongation in the maintenance of genome stability in metazoans.

STAR★METHODS

Detailed methods are provided in the online version of this paper and include the following:

- KEY RESOURCES TABLE
- RESOURCE AVAILABILITY
  - Lead Contact
  - Materials Availability
  - Data and Code Availability
- EXPERIMENTAL MODEL AND SUBJECT DETAILS
  - Mouse strains and cell lines
  - *C. elegans* strains
- METHOD DETAILS
  - RNA interference and plate phenotype scoring
  - Immunofluorescence analysis in C.elegans
  - Assay for poly-G/C-tract deletions
  - Mouse Embryonic Fibroblasts (MEFs) isolation and culture

Figure 6. RTEL1-POLE4 Double Knockout Cells Accumulate DNA Damage and Genomic Instability

(A) Top: bar graphs showing percentage of cells with more than 10 γH2AX foci from *RTEL1*$^{F/F}$ *POLE4*$^{+/+}$ and *RTEL1*$^{F/F}$ *POLE4*$^{-/-}$ MEFs infected with GFP-CRE or empty GFP. Cells were analyzed 72 h after infection. Bars represent mean ± SD of triplicate experiments (***p < 0.001, ****p < 0.0001). Bottom: representative immunofluorescence staining from *RTEL1*$^{F/F}$ *POLE4*$^{+/+}$ and *RTEL1*$^{F/F}$ *POLE4*$^{-/-}$ MEFs infected with GFP-CRE or empty GFP.

(B) Top: bar graphs showing percentage of cells with more than 5 53BP1 foci from *RTEL1*$^{F/F}$ *POLE4*$^{+/+}$ and *RTEL1*$^{F/F}$ *POLE4*$^{-/-}$ MEFs infected with GFP-CRE or empty GFP. Cells were analyzed 72 h after infection. Bars represent mean ± SD of triplicate experiments. (*p < 0.05, **p < 0.01). Bottom: representative immunofluorescence staining from *RTEL1*$^{F/F}$ *POLE4*$^{+/+}$ and *RTEL1*$^{F/F}$ *POLE4*$^{-/-}$ MEFs infected with GFP-CRE or empty GFP.

(C) Left: bar graphs showing percentage of cells with micronuclei; bars represent mean ± SD of triplicate experiments (***p < 0.001). Right: representative images of micronuclei in cells of the indicated genotype. Scale bar, 10 μm.

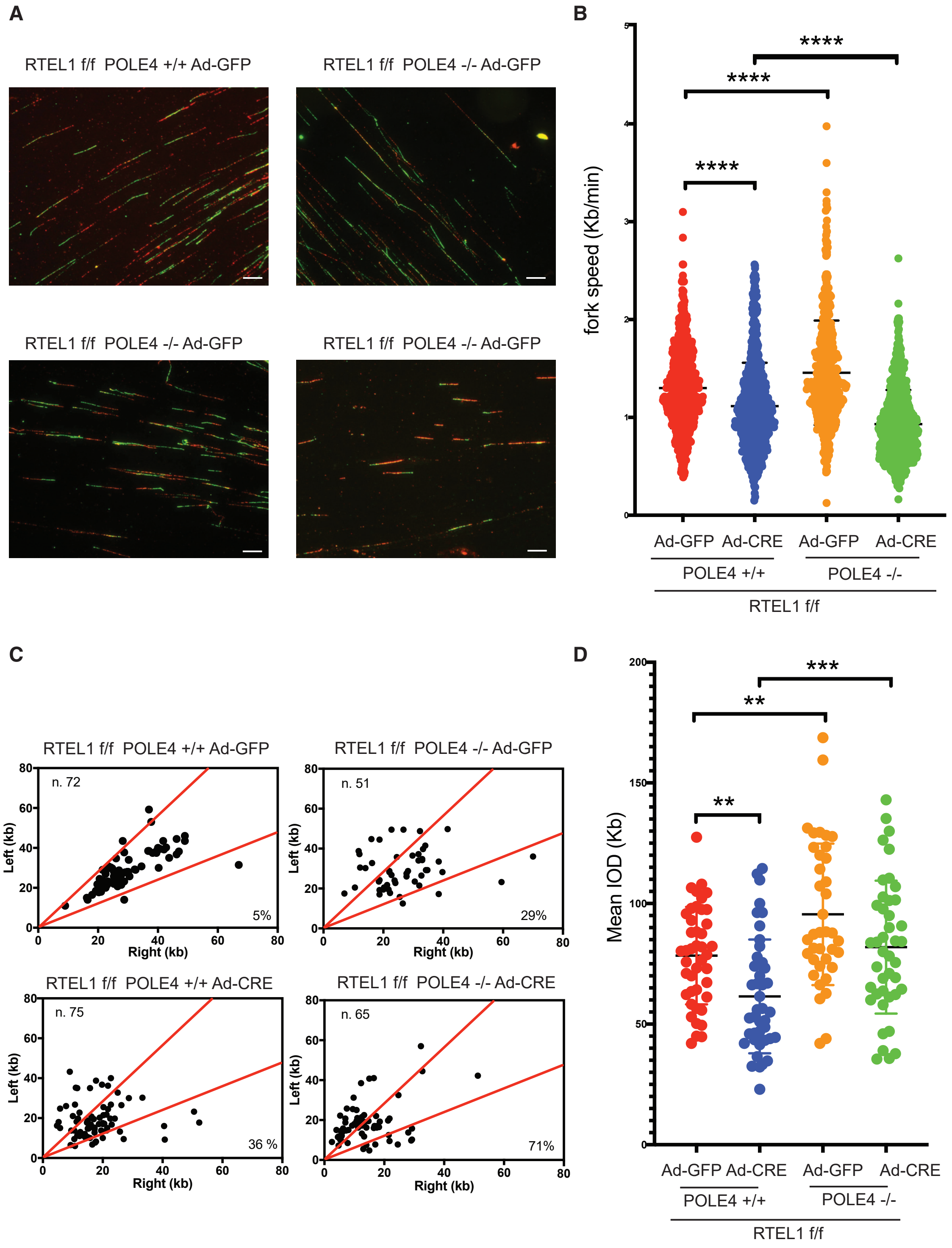

Figure 7. Impaired Fork Progression and Increased Fork Stalling in RTEL1-POLE4 Double Knockout Cells

(A) Representative DNA fiber immunofluorescence from *RTEL1*$^{F/F}$ *POLE4*$^{+/+}$ and *RTEL1*$^{F/F}$ *POLE4*$^{-/-}$ MEFs infected with GFP-CRE or empty GFP.

(B) Bar graphs showing replication fork speed (measured as IdU track length/min) from *RTEL1*$^{F/F}$ *POLE4*$^{+/+}$ and *RTEL1*$^{F/F}$ *POLE4*$^{-/-}$ MEFs transduced or not with CRE. ****$p < 0.0001$.

(C). Analysis of replication fork symmetry from *RTEL1*$^{F/F}$ *POLE4*$^{+/+}$ and *RTEL1*$^{F/F}$ *POLE4*$^{-/-}$ MEFs infected with GFP-CRE or empty GFP. Total number of newly established replication forks analyzed is indicated.

(D) Bar graphs showing inter-origin distance measurements from *RTEL1*$^{F/F}$ *POLE4*$^{+/+}$ and *RTEL1*$^{F/F}$ *POLE4*$^{-/-}$ MEFs transduced or not with CRE recombinase (**$p < 0.01$, ***$p < 0.001$). Error bars represent standard deviation (SD) of the mean. Scale bars, 10 μm.

- ○ Cre-mediated recombination
- ○ Immunofluorescence staining
- ○ DNA fiber stretching assay
- QUANTIFICATION AND STATISTICAL ANALYSES

SUPPLEMENTAL INFORMATION

Supplemental Information can be found online at https://doi.org/10.1016/j.celrep.2020.107675.

ACKNOWLEDGMENTS

We would like to thank the Francis Crick Biological Resource facility for assistance with mouse breeding and maintenance and Joe Brock for help with figure generation. R.B. and V.B. are funded by the Francis Crick Institute. Boulton lab work is supported by the Francis Crick Institute, which receives its core funding from Cancer Research UK (FC0010048), the UK Medical Research Council (FC0010048), and the Wellcome Trust (FC0010048); a European Research Council (ERC) Advanced Investigator Grant (TelMetab); and Wellcome Trust senior investigator and collaborative grants.

AUTHOR CONTRIBUTIONS

S.J.B. conceived the project. J.Y. performed the RNAi screening in *C. elegans* and experiments in worms with help from J.S. and V.T.-P. R.B. performed experiments in mammalian cells with help from V.B. R.B., J.Y., and S.J.B. wrote the paper.

DECLARATION OF INTERESTS

S.J.B. is scientific co-founder and SVP Science Strategy at Artios Pharma Ltd., Babraham, UK.

Received: February 20, 2020
Revised: March 28, 2020
Accepted: April 30, 2020
Published: May 26, 2020

REFERENCES

Barber, L.J., Youds, J.L., Ward, J.D., McIlwraith, M.J., O'Neil, N.J., Petalcorin, M.I., Martin, J.S., Collis, S.J., Cantor, S.B., Auclair, M., et al. (2008). RTEL1 maintains genomic stability by suppressing homologous recombination. Cell *135*, 261–271.

Bell, S.P., and Labib, K. (2016). Chromosome Duplication in *Saccharomyces cerevisiae*. Genetics *203*, 1027–1067.

Bellelli, R., Borel, V., Logan, C., Svendsen, J., Cox, D.E., Nye, E., Metcalfe, K., O'Connell, S.M., Stamp, G., Flynn, H.R., et al. (2018). Polε Instability Drives Replication Stress, Abnormal Development, and Tumorigenesis. Mol. Cell *70*, 707–721.e7.

Bhat, K.P., and Cortez, D. (2018). RPA and RAD51: fork reversal, fork protection, and genome stability. Nat. Struct. Mol. Biol. *25*, 446–453.

Burgers, P.M.J., and Kunkel, T.A. (2017). Eukaryotic DNA Replication Fork. Annu. Rev. Biochem. *86*, 417–438.

Cheung, I., Schertzer, M., Rose, A., and Lansdorp, P.M. (2002). Disruption of dog-1 in *Caenorhabditis elegans* triggers deletions upstream of guanine-rich DNA. Nat. Genet. *31*, 405–409.

Dehé, P.M., and Gaillard, P.H.L. (2017). Control of structure-specific endonucleases to maintain genome stability. Nat. Rev. Mol. Cell Biol. *18*, 315–330.

Dungrawala, H., Rose, K.L., Bhat, K.P., Mohni, K.N., Glick, G.G., Couch, F.B., and Cortez, D. (2015). The Replication Checkpoint Prevents Two Types of Fork Collapse without Regulating Replisome Stability. Mol. Cell *59*, 998–1010.

Fragkos, M., Ganier, O., Coulombe, P., and Méchali, M. (2015). DNA replication origin activation in space and time. Nat. Rev. Mol. Cell Biol. *16*, 360–374.

Ge, X.Q., Jackson, D.A., and Blow, J.J. (2007). Dormant origins licensed by excess Mcm2-7 are required for human cells to survive replicative stress. Genes Dev. *21*, 3331–3341.

Hustedt, N., Álvarez-Quilón, A., McEwan, A., Yuan, J.Y., Cho, T., Koob, L., Hart, T., and Durocher, D. (2019). A consensus set of genetic vulnerabilities to ATR inhibition. Open Biol. *9*, 190156.

Ibarra, A., Schwob, E., and Méndez, J. (2008). Excess MCM proteins protect human cells from replicative stress by licensing backup origins of replication. Proc. Natl. Acad. Sci. USA *105*, 8956–8961.

Kamath, R.S., Fraser, A.G., Dong, Y., Poulin, G., Durbin, R., Gotta, M., Kanapin, A., Le Bot, N., Moreno, S., Sohrmann, M., et al. (2003). Systematic functional analysis of the *Caenorhabditis elegans* genome using RNAi. Nature *421*, 231–237.

León-Ortiz, A.M., Svendsen, J., and Boulton, S.J. (2014). Metabolism of DNA secondary structures at the eukaryotic replication fork. DNA Repair (Amst.) *19*, 152–162.

Muramatsu, S., Hirai, K., Tak, Y.S., Kamimura, Y., and Araki, H. (2010). CDK-dependent complex formation between replication proteins Dpb11, Sld2, Pol (epsilon, and GINS in budding yeast. Genes Dev. *24*, 602–612.

Navas, T.A., Zhou, Z., and Elledge, S.J. (1995). DNA polymerase epsilon links the DNA replication machinery to the S phase checkpoint. Cell *80*, 29–39.

Panier, S., and Boulton, S.J. (2014). Double-strand break repair: 53BP1 comes into focus. Nat. Rev. Mol. Cell Biol. *15*, 7–18.

Saito, T.T., Youds, J.L., Boulton, S.J., and Colaiácovo, M.P. (2009). *Caenorhabditis elegans* HIM-18/SLX-4 interacts with SLX-1 and XPF-1 and maintains genomic integrity in the germline by processing recombination intermediates. PLoS Genet *5*, e1000735.

Sarek, G., Vannier, J.B., Panier, S., Petrini, J.H.J., and Boulton, S.J. (2015). TRF2 recruits RTEL1 to telomeres in S phase to promote t-loop unwinding. Mol. Cell *57*, 622–635.

Sparks, J.L., Chistol, G., Gao, A.O., Räschle, M., Larsen, N.B., Mann, M., Duxin, J.P., and Walter, J.C. (2019). The CMG Helicase Bypasses DNA-Protein Cross-Links to Facilitate Their Repair. Cell *176*, 167–181.e21.

Técher, H., Koundrioukoff, S., Nicolas, A., and Debatisse, M. (2017). The impact of replication stress on replication dynamics and DNA damage in vertebrate cells. Nat. Rev. Genet. *18*, 535–550.

Vannier, J.B., Pavicic-Kaltenbrunner, V., Petalcorin, M.I., Ding, H., and Boulton, S.J. (2012). RTEL1 dismantles T loops and counteracts telomeric G4-DNA to maintain telomere integrity. Cell *149*, 795–806.

Vannier, J.B., Sandhu, S., Petalcorin, M.I., Wu, X., Nabi, Z., Ding, H., and Boulton, S.J. (2013). RTEL1 is a replisome-associated helicase that promotes telomere and genome-wide replication. Science *342*, 239–242.

Ward, J.D., Barber, L.J., Petalcorin, M.I., Yanowitz, J., and Boulton, S.J. (2007). Replication blocking lesions present a unique substrate for homologous recombination. EMBO J. *26*, 3384–3396.

Youds, J.L., O'Neil, N.J., and Rose, A.M. (2006). Homologous recombination is required for genome stability in the absence of DOG-1 in *Caenorhabditis elegans*. Genetics *173*, 697–708.

Wicky, C., Alpi, A., Passanante, M., Rose, A., Gartner, A., and Müller, F. (2004). Multiple genetic pathways involving the *Caenorhabditis elegans* Bloom's syndrome genes *him-6*, *rad-51*, and *top-3* are needed to maintain genome stability in the germ line. Mol. Cell. Biol. *24*, 5016–5017.

Youds, J.L., Barber, L.J., Ward, J.D., Collis, S.J., O'Neil, N.J., Boulton, S.J., and Rose, A.M. (2008). DOG-1 is the *Caenorhabditis elegans* BRIP1/FANCJ homologue and functions in interstrand cross-link repair. Mol. Cell. Biol. *28*, 1470–1479.

Youds, J.L., Mets, D.G., McIlwraith, M.J., Martin, J.S., Ward, J.D., ONeil, N.J., Rose, A.M., West, S.C., Meyer, B.J., and Boulton, S.J. (2010). RTEL-1 enforces meiotic crossover interference and homeostasis. Science *327*, 1254–1258.

Zeman, M.K., and Cimprich, K.A. (2014). Causes and consequences of replication stress. Nat. Cell Biol. *16*, 2–9.

STAR★METHODS

KEY RESOURCES TABLE

REAGENT or RESOURCE	SOURCE	IDENTIFIER
Antibodies		
Goat Anti-Rat IgG (H+L) Antibody, Alexa Fluor 594 Conjugated	Thermo Fisher	Cat#A-11007; RRID: AB_141374
Rabbit Anti-Mouse IgG (H+L) Antibody, Alexa Fluor488 Conjugated	Thermo Fisher	Cat#A-11059; RRID: AB_142495
Goat Anti-Rabbit IgG (H+L) Antibody, Alexa Fluor488 Conjugated	Thermo Fisher	Cat#A-11034
Rabbit polyclonal anti-53BP1	Novus Biologicals	Cat#NB100-304; RRID: AB_10003037
Mouse monoclonal γH2AX clone JBW301	Millipore	Cat#05-63; RRID: AB_309864
Rat monoclonal anti-BrdU	AbD Serotec	Cat#OBT0030
Mouse monoclonal anti-BrdU	Becton Dickinson	Cat#347580
Chemicals, Peptides, and Recombinant Proteins		
Adenovirus Ad-Cre-GFP	Vector Biolabs	Cat#1700
Adenovirus Ad-GFP	Vector Biolabs	Cat#1060
CldU	Sigma-Aldrich	Cat#C6891
IdU	Sigma-Aldrich	Cat#I7125
EdU	Thermo Fisher Scientific	Cat#A10044
Benzonase	Novagen	Cat#71206-3
DAPI	SIGMA	Cat#10236276001
Critical Commercial Assays		
FiberPrep® (DNA Extraction Kit)	Genomic Vision	Cat#EXTR-001
Lipofectamine 2000	Thermo Fisher	Cat# 11668027
QIAprep Spin Miniprep Kit	QIAGEN	Cat#27106
Click-iT EdU Alexa Fluor 488 Flow Cytometry Assay Kit	Thermo Fisher	Cat#C10425
Experimental Models: Mouse Strains		
$Pole4^{tm1(KOMP)Vlcg}$	Bellelli et al., 2018	N/A
RTEL1 f/f	Vannier et al., 2012	N/A
Experimental Models: Cell Lines		
Mouse Embryonic Fibroblasts $RTEL1^{f/f}$ $Pole4^{+/+}$	This study	N/A
Mouse Embryonic Fibroblasts $RTEL1^{f/f}$ $Pole4^{-/-}$	This study	N/A
Human HEK293 cells	The Francis Crick Institute Cell Services	N/A
Experimental Models: C. elegans strains		
C. elegans: WT, Bristol (N2) background	CGC	N2
FX1866 *rtel-1(tm1866)*	Barber et al., 2008	DW663
FX4613 *Y53F4B.3 pole-4(tm4613)*	CGC	N/A
FX1937 *mus-81(tm1937)*	Barber et al., 2008	N/A
DW238 *rtel-1(tm1866) mus-81(tm1937)*/hT2[*bli-4(e937) let-?(q782)* qIs48](I;III)	This study	N/A
VC13 *dog-1(gk10)*	Cheung et al., 2002	N/A
VC193 *him-6(ok412)*,	Wicky et al., 2004	N/A
RB1279 *rfs-1(ok1372)*	Ward et al., 2007	N/A
CB1487 *him-9(e1487)*	Saito et al., 2009	N/A
Software and Algorithms		
Adobe Photoshop CC	Adobe	https://www.adobe.com/es/products/photoshop.html

(*Continued on next page*)

Continued

REAGENT or RESOURCE	SOURCE	IDENTIFIER
ImageJ	NIH	https://imagej.nih.gov/ij/
Volocity 6.3	PerkinElmer	http://www.perkinelmer.com/lab-products-and-services/resources/cellular-imaging-software-downloads.html
GraphPad Prism 7	GraphPad	https://www.graphpad.com/

RESOURCE AVAILABILITY

Lead Contact

Further information and requests for reagents should be directed to and will be fulfilled by the Lead Contact, Simon Boulton (simon.boulton@crick.ac.uk).

Materials Availability

Mouse cell lines and *C. elegans* strains generated in this study are available upon request to the Lead Contact (simon.boulton@crick.ac.uk).

Data and Code Availability

This study did not generate/analyze datasets/code.

EXPERIMENTAL MODEL AND SUBJECT DETAILS

Mouse strains and cell lines

Mouse strains and cell lines used in the study are listed in Key Resource Table. Mouse Embryonic Fibroblasts were produced at embryonic day 13.5 from timed breeding between 8-12 weeks old *RTEL1$^{fl/fl}$ Pole4$^{+/-}$* males and females. All animal experimentations were undertaken in compliance with UK Home Office legislation (project license number 70/8527) under the Animals (Scientific Procedures) Act 1986. *Rtel1$^{F/F}$ Pole4$^{+/+}$ and Pole4$^{-/-}$* primary mouse embryonic fibroblasts (MEFs) were cultured at 37°C/ 5% CO_2/ 5% O_2 in Dulbecco's modified Eagle's medium (DMEM) (Invitrogen) supplemented with 15% fetal bovine serum (Sigma) and 1% penicillin-streptomycin (Invitrogen). 293 cells were cultured in 37°C/ 5% CO_2/ 5% O_2 in Dulbecco's modified Eagle's medium (DMEM) (Invitrogen) supplemented with 15% fetal bovine serum (Sigma) and 1% penicillin-streptomycin (Invitrogen). Human 293 cells were cultured in DMEM 10% FBS (SIGMA) at 37°C/ 5% CO_2.

C. elegans strains

The strains used in this work are listed in Key Resource Table and include FX1866 *rtel-1(tm1866)*, FX4613 *Y53F4B.3 pole-4(tm4613)*, FX1937 *mus-81(tm1937)*, DW238 *rtel-1(tm1866) mus-81(tm1937)*/hT2[*bli-4(e937) let-?(q782)* qIs48](I;III), VC13 *dog-1(gk10)*, VC193 *him-6(ok412)*, RB1279 *rfs-1(ok1372)* and CB1487 *him-9(e1487)*. *rtel-1*, *pole-4* and *mus-81* deletion strains were kindly provided by Shohei Mitani and the National Bio-resource Project. Strains were also obtained from the *Caenorhabditis* Genetics Centre and outcrossed to N2 multiple times before use. Strain maintenance and new strain construction was carried out by standard methods.

METHOD DETAILS

RNA interference and plate phenotype scoring

Secondary RNAi screen and scoring data was collected by feeding worms with RNAi bacteria on 55mm MYOB plates. Clones of interest were located in a previously described RNAi library and streaked onto LB agar + Ampicillin 50ug/mL plates and grown overnight at 37 degrees. Single colonies were grown overnight at 37 degrees in 5mL of LB + Ampicillin 50ug/mL. Cultures were centrifuged and resuspended in one-third of the original volume. For each RNAi plate, 50uL of RNAi bacteria was spotted onto MYOB plates containing 50ug/mL Ampicillin and 1mM IPTG. Plates were incubated at room temperature overnight before use. L1-stage worms were plated onto the RNAi plates and were transferred to fresh RNAi plates each day after the start of egg laying for 4 days. For scoring of double mutant strains and controls (under no RNAi conditions), L4-stage animals were individually plated and transferred onto fresh plates each day for 4 days. Unhatched eggs were scored 24 hours after removing the parent animal from the plate, and the total number of viable progeny and males (if scored) was counted after an additional 24-48 hours.

Immunofluorescence analysis in C.elegans

Germlines of young adult animals were extracted and fixed in 4% PFA, and permeated by incubation with TBS containing 0.5% BSA and 0.1% Triton X-100. Rabbit anti-RPA and rabbit anti-RAD-51 primary antibodies were both used at 1:500 dilutions in TBS + 0.5% BSA and incubated overnight at 4 degrees. Alexa 488 goat anti-rabbit secondary antibody (Invitrogen) was used at 1:10000 dilution in TBS + 0.5% BSA. Germlines were also stained with DAPI (Sigma) and slides were mounted using Vectashield (Vector Labs). Slides were viewed on a Deltavision microscope (Appiled Precision) with 100X lens. Images were deconvolved using SoftWoRX software. RAD-51 foci in individual nuclei were counted using the Z stacks of images.

Assay for poly-G/C-tract deletions

The assay for poly-G/C-tract deletions was carried out as described in Youds et al. (2006).

Mouse Embryonic Fibroblasts (MEFs) isolation and culture

RTEL1$^{fl/fl}$ *Pole4*$^{+/-}$ mice in C57BL/6 background were mated. Pregnant females at 13.5 days gestation were subjected to euthanasia under anesthesia, followed by uterine dissection to isolate individual embryos. After washing in PBS and removal of head (used for embryo genotyping) and internal organs (heart and liver), embryo bodies were minced with sterile razor blades and incubated in trypsin at 37°C for 10 min, followed by gentle pipetting of the trypsin digest. Cell suspension was pelleted, resuspended and plated in 10 cm dishes (passage 0) in DMEM (Dulbecco's modified Eagle's medium (DMEM) supplemented with 15% FBS (SIGMA) and 50μg/ml penicillin-streptomycin, 2mM L-glutamine. Once subconfluent, a standard 3T3 protocol was followed: every 3 days cells were trypsinized, counted using cellometer Auto 2000 (Nexcelom Bioscience) to determine the number of Population doublings (PD) and then re-plated at a fixed density ($8x10^5$ cells per 100-mm dish) The accumulation of population doubling level (PDL) was calculated using the formula $\Delta PDL = log(nh/ni)/log2$, where ni is the initial number of cells and nh is the cell number at each passage.

Cre-mediated recombination

Rtel1$^{F/F}$ *Pole4*$^{+/+}$ *and Pole4*$^{-/-}$ mouse primary cells were infected with adenovirus expressing the CRE recombinase together with a GFP marker to inactivate *Rtel1* (Ad-CRE) or control adenovirus expressing only GFP (Ad-GFP). Samples were processed for analysis 72 hours after infection and loss of RTEL1 was verified by PCR and/or western blot (Sarek et al., 2015).

Immunofluorescence staining

For indirect immunofluorescence staining, cells were seeded on coverslips and fixed in 4% paraformaldehyde. After permeabilization with 0.5% Triton X-100 (5 min on ice), coverslips were blocked in 1% BSA/PBS and incubated with the following primary antibodies diluted in 0.5% BSA/PBS: anti-H2AX phosphorylated on Ser139 (γH2AX) (Millipore), −53BP1, (Novus Biologicals), for 1h at room temperature. Coverslips were then washed 3 times in PBS and incubated with Alexa Fluor 488 goat anti-rabbit or rabbit anti-mouse antibodies (Invitrogen) for 45 min at room temperature. After DAPI counterstaining, coverslips were mounted in Glycerol/PBS (1:1) and observed with Axio Imager.M2 (ZEISS) using the Volocity 6.3 software. For EdU immunofluorescence analysis MEFs (passage 3) were labeled and processed using the Click-iT® EdU Flow Cytometry Cell Proliferation Assay (Thermo Fisher). Cells were pulse labeled for 30 min with 10 mM EdU and fixed in 4% paraformaldehyde, before being permeabilized in PBS-Triton 0.5% and washed in 1% BSA. Cells were then resuspended in Click-iT reaction cocktail containing Alexa Fluor® 488 Azide and incubated for 30 min at R.T. After being washed, cells were finally counterstained for DNA content by DAPI (1 mg/ml) and analyzed using a Flow cytometry analyzer LSRII (Becton Dickinson).

DNA fiber stretching assay

DNA fiber assay was performed as described in Bellelli et al. (2018). Briefly, *Rtel1*$^{F/F}$ *Pole4*$^{+/+}$ *and Pole4*$^{-/-}$ MEFs infected with CRE recombinase or GFP expressing adenovirus, were pulse labeled with 20 μM CldU for 20 min and subsequently with 200 μM IdU for 20 min. Cells were trypsinized, washed in PBS and resuspended at a concentration of $5x\ 10^5$ in PBS. 2.5 μL of cell suspension were spotted on clean glass slides and lysed with 7.5 μL of 0.5% SDS in 200 mM Tris-HCL, pH 7.4, 50 mM EDTA for 10 min at R.T. Slides were then tilted allowing a stream of DNA to run slowly down the slide, air-dried and then fixed in methanol/acetic acid (3:1) for 15 min at R.T. After denaturation in HCl 2,5 M (30 min R.T.) slides were blocked in 1% BSA/PBS and incubated with rat anti-BrdU monoclonal antibody (1:1000 overnight; AbD Serotec) and mouse anti-BrdU monoclonal antibody (1:500 1h R.T.; Becton Dickinson). After washes in PBS, slides were incubated with Alexa Fluor 488 rabbit anti-mouse and Alexa Fluor 594 goat anti-rat antibodies (1:500 R.T.; Invitrogen) for 45 min and mounted in PBS/Glycerol 1:1. Fibers were then examined using Axio Imager.M2 (ZEISS) with 60x oil immersion objective and the Volocity 6.3 software.

QUANTIFICATION AND STATISTICAL ANALYSES

Statistics, including statistical tests used, number of events quantified, standard deviation standard error of the mean, and statistical significance are reported in figures and figure legends. Statistical analysis has been performed using GraphPad Prism7 software (GraphPad) and statistical significance is determined by the value of $p < 0.05$.

Cell Reports, Volume *31*

Supplemental Information

Synthetic Lethality between DNA Polymerase Epsilon and RTEL1 in Metazoan DNA Replication

Roberto Bellelli, Jillian Youds, Valerie Borel, Jennifer Svendsen, Visnja Pavicic-Kaltenbrunner, and Simon J. Boulton

Supplemental Figure S1

A

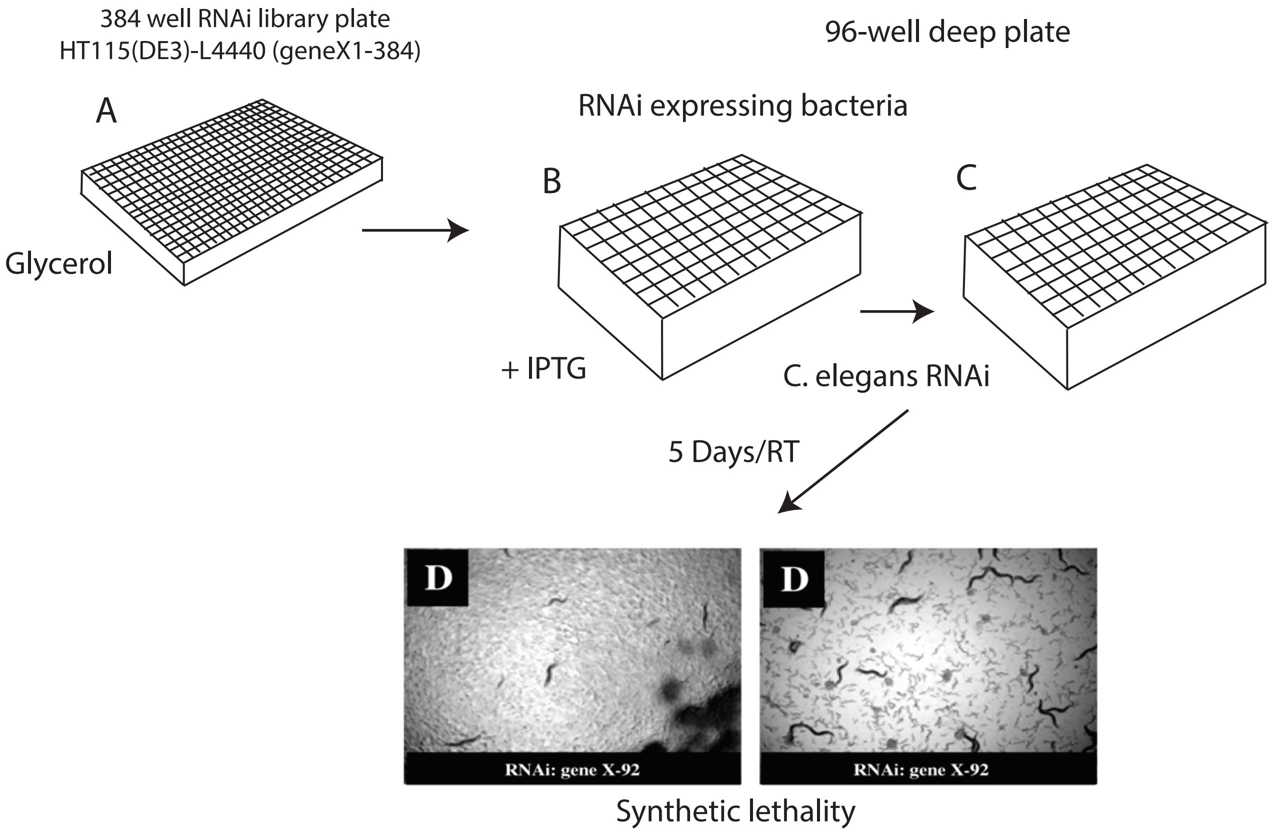

B

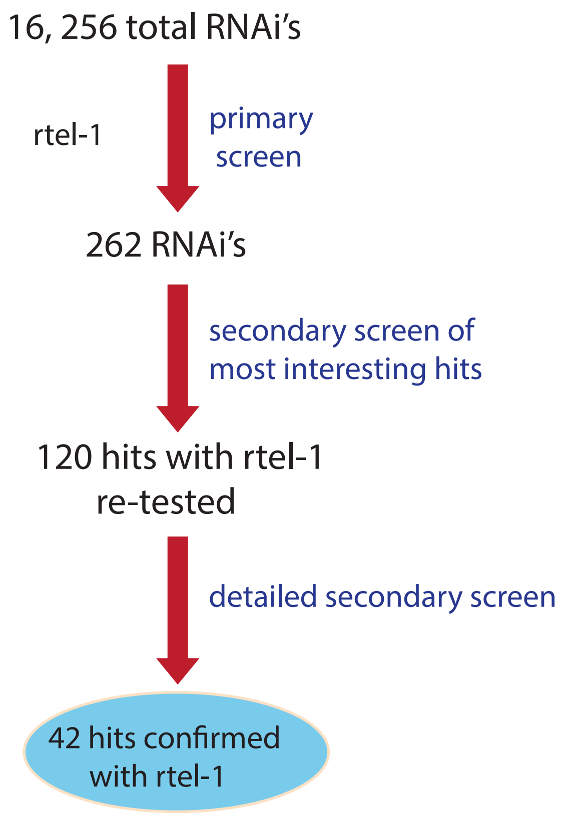

C

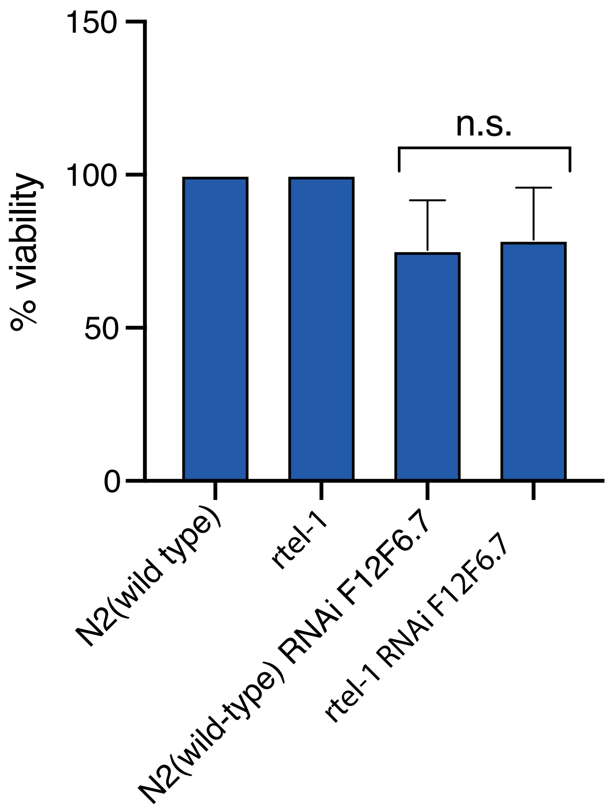

Figure S1 (related to Figure 1). A synthetic viability screening in *rtel-1* mutant C. elegans animals. (A) Representative scheme of the RNAi screening performed in *rtel-1* wt and mutant worms. (B) Diagram showing the sequential primary and secondary RNAi screenings performed in *rtel-1* wt and mutant strains and the confirmed hits (C). Percent viability after feeding either no RNAi or RNAi for *pold-2* (F12F6.7) in wild type or *rtel-1* mutant worms. percent viability is normalized based on untreated wild type or *rtel-1* control animals (n.s. not significant).

Supplemental Figure S2

A

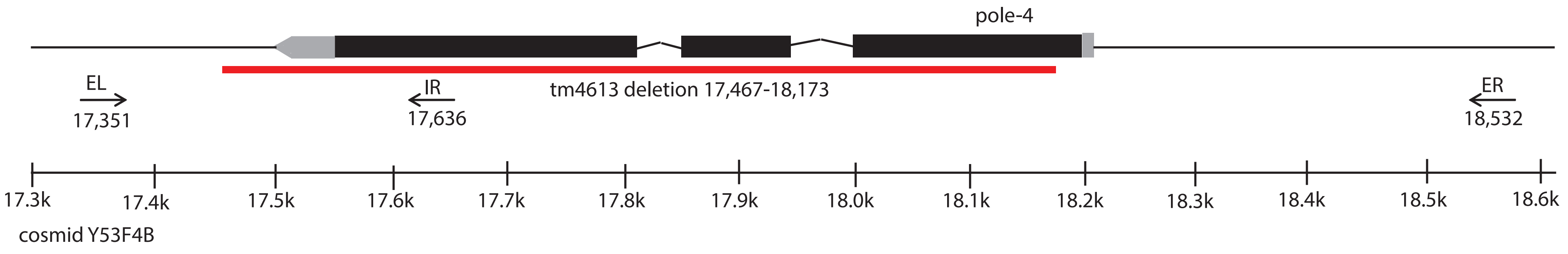

B

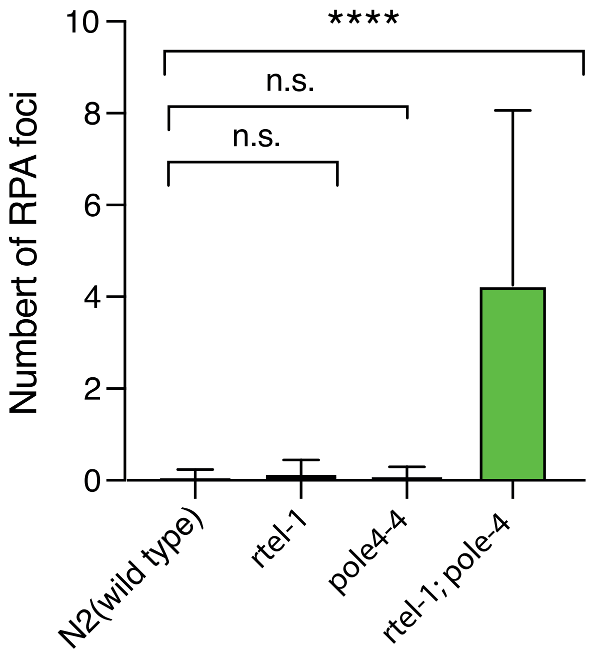

Figure S2 (related to Figure 2). (A) Schematic diagram of C. elegans *Y53F4B.3* (*pole-4*) gene. The genetic deletion identified in the *tm4613* strain is highlighted with a red bar. (B). Bar-graphs showing the average number of RPA foci +/- SD in the indicated genotypes (**** p <0.0001, n.s. not significant).

Supplemental Figure S3

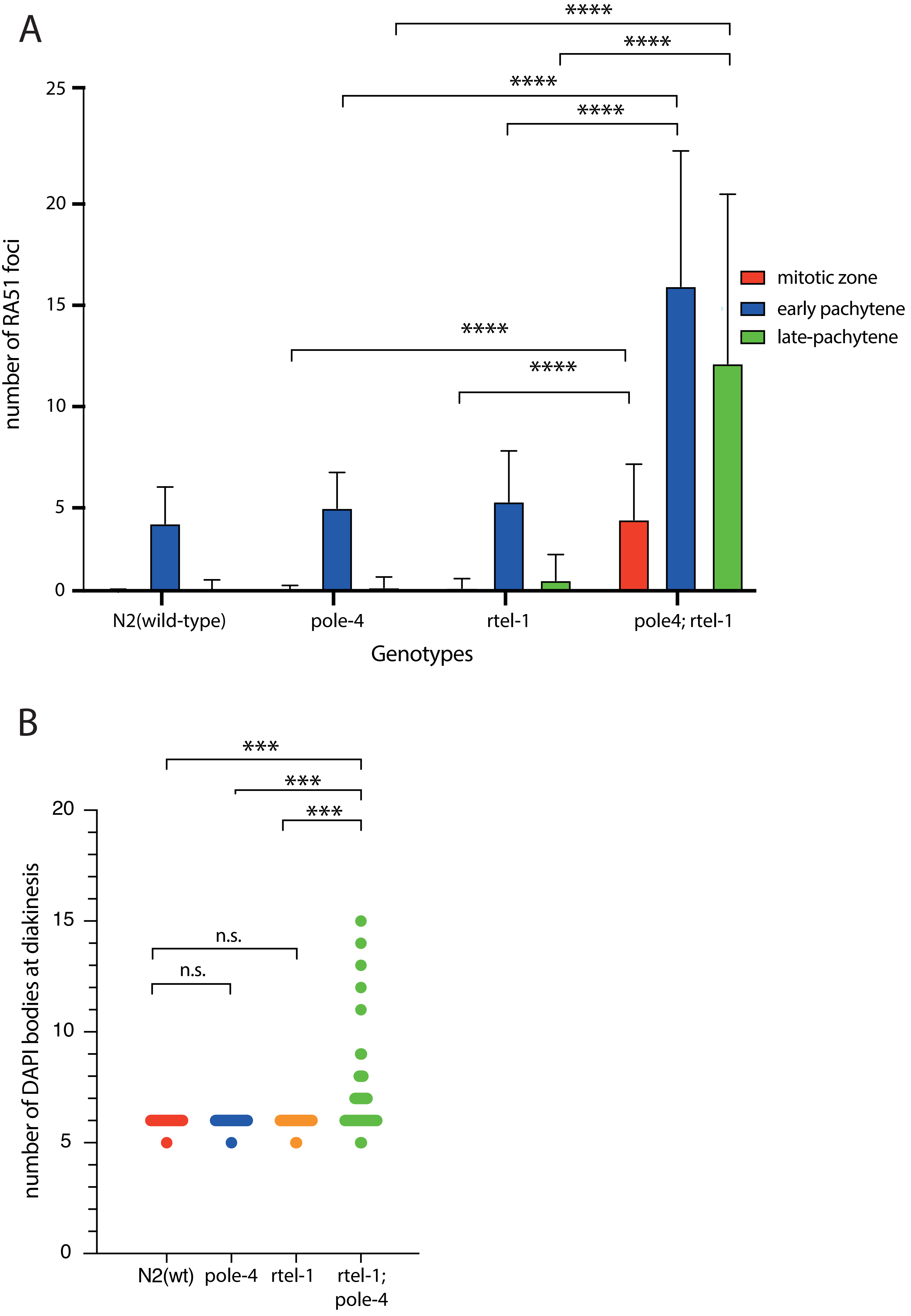

Figure S3 (related to Figure 3). (A) Bar-graphs showing the number of RAD51 foci in the defined zones of the germline in the indicated genotypes (**** $p < 0.0001$). (B). Bar-graphs showing the number of DAPI bodies at diakinesis in the indicated genotypes (*** $p < 0.001$, n.s. not significant).

Supplemental Table S1

Genotype	Brood Size	Percent Survival	Percent Males (of viable)
**wild type**	299 ±	99.6 ± 0.1	0.1 ± 0.1
pole-4 (tm4613)	237 ±	99.8 ± 0.1	0.0 ± 0.0
dog-1 (gk10)	300 ± 28	99.7 ± 0.2	0.1 ± 0.1
rtel-1 (tm1866)	61 ± 16	92.6 ± 3.6	0.0 ± 0.0
mus-81 (tm1937)	114 ± 19	90.1 ± 3.7	0.1 ± 0.1
him-6 (ok412)	221 ± 13	48.3 ± 3.2	11.7 ± 0.8
rcq-5(tm424)	292 ± 32	99.4 ± 1.0	0.1 ± 0.1
rtel-1; pole-4	29 ±	0	N/A
dog-1; pole-4	198 ± 15	91.6 ± 5.1	0.3 ± 0.1
mus-81; pole-4	107 ± 9	96.7 ± 1.0	0.6 ± 0.3
pole-4; him-6	123 ± 15	48.2 ± 3.3	11.7 ± 1.0
pole-4; rcq-5	227 ± 22	99.4 ± 0.2	0.0 ± 0.0
rtel-1 mus-81; pole-4	0	0	N/A
rtel-1; pole-4; rfs-1	20 ± 7	0	N/A

Supplemental Table S1 (related to Figure 4). Genetic interactions of *pole-4*. Brood size indicates the average total number of eggs laid by individual animals. Percent survival indicates the percent of the total brood that survived to adulthood. For each genotype, the progeny of at least 10 parent animals were scored.

Supplemental Table S2

Genotype	Animals Tested	Animals with Deletions	Percent with Deletions
**wild type**	120	0	0
dog-1(gk10)	192	15	7.8
pole-2(RNAi)	120	0	0
dog-1; pole-2(RNAi)	228	18	7.9
rtel-1(tm1866)	92	0	0
pole-4 (tm4613)	96	0	0
rtel-1; pole-4	96	0	0

Supplemental Table S2 (related to Figure 4). Poly-G-tracts are stable in polymerase epsilon mutants and in *rtel-1; pole-4* animals. The presence of G-tract deletions in the genotypes indicated was measured by nested PCR at the G-tract within the *vab-1* gene.

Supplemental Figure S4

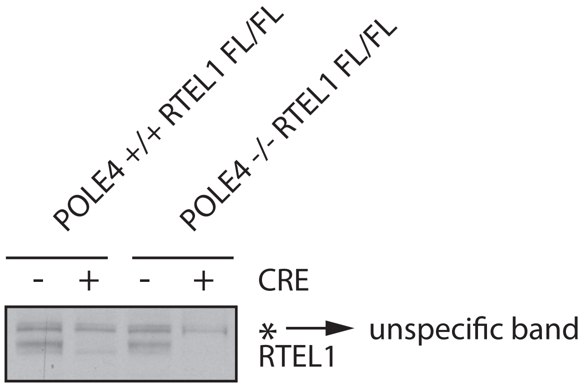

Figure S4 (related to Figure 5). Western blot analysis of RTEL1 expression levels in RTEL1$^{F/F}$ *POLE4*$^{+/+}$ and RTEL1$^{F/F}$ *POLE4*$^{-/-}$ MEFs infected with GFP-CRE or empty GFP. An unspecific band (*) served as loading control.

Supplemental Figure S5

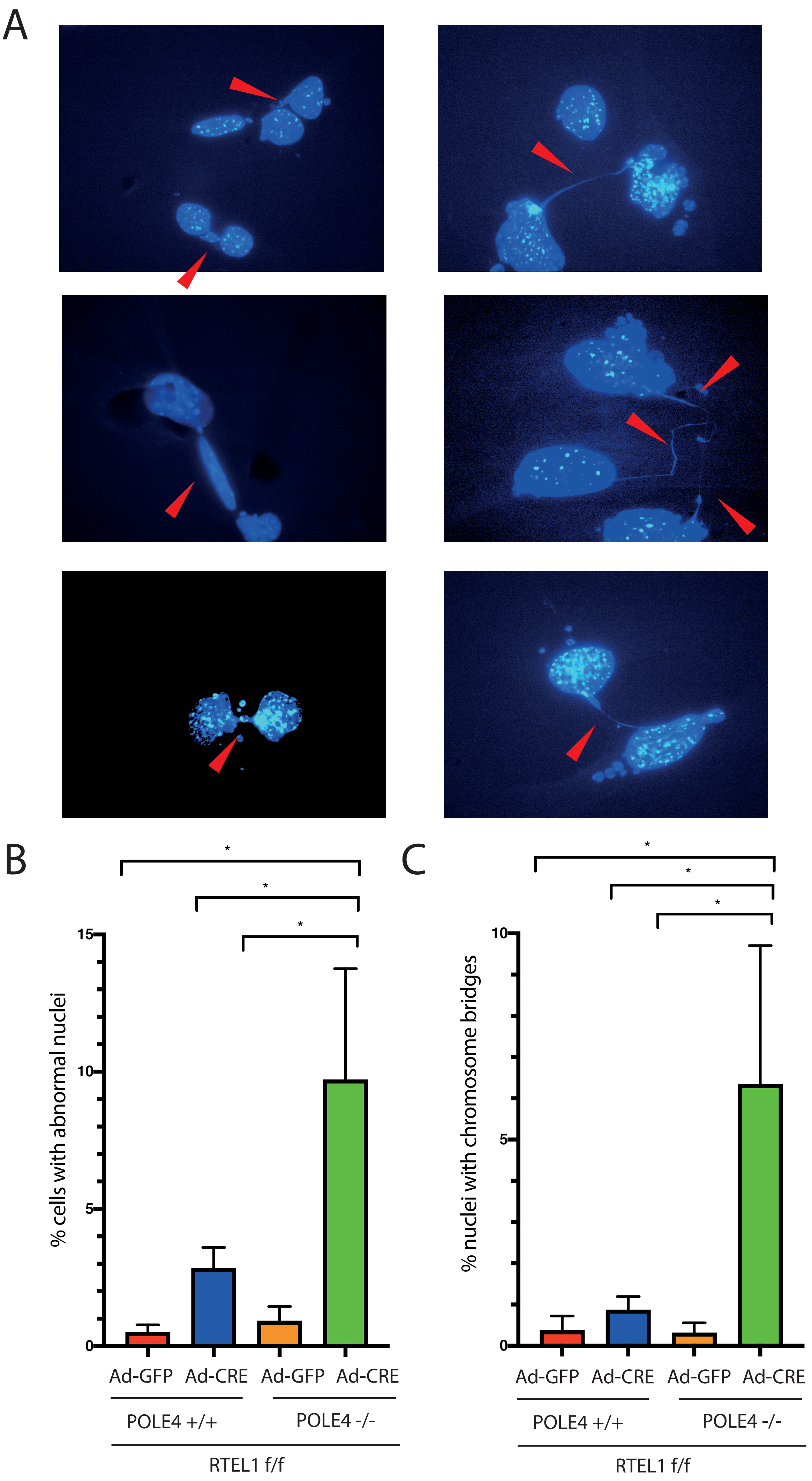

Figure S5 (related to Figure 6). (A) Representative images of nuclear aberrations observed in RTEL1$^{F/F}$ *POLE*$^{-/-}$ MEFs infected with GFP-CRE. Red arrows indicate mitotic bridges. (B) Bar-graphs showing percentage of cells with abnormal nuclear structures from RTEL1$^{F/F}$ *POLE4*$^{+/+}$ and RTEL1$^{F/F}$ *POLE4*$^{-/-}$ infected or not with CRE (* $p< 0.05$). (C) Bar-graphs showing percentage of nuclei with chromosome bridges from RTEL1$^{F/F}$ *POLE4*$^{+/+}$ and RTEL1$^{F/F}$ *POLE4*$^{-/-}$ infected or not with CRE (* $p< 0.05$).
